# Supplementary material for: Molecular architecture of chitin and chitosan-dominated cell walls in zygomycetous fungal pathogens by solid-state NMR
Source: Nat Commun. 2024 Sep 27;15:8295. doi: 10.1038/s41467-024-52759-8 (PMC11437000; doi:10.1038/s41467-024-52759-8)
Supplement: Supplementary file 1 — Supplementary Information [file 41467_2024_52759_MOESM1_ESM.pdf]

Supplementary Information

Molecular Architecture of Chitin and Chitosan-Dominated Cell Walls in Zygomycetous Fungal Pathogens by Solid-State NMR

Qinghui Cheng[1, ‡], Malitha C. Dickwella Widanage[1, ‡, †], Jayasubba Reddy Yarava[1], Ankur Ankur[1], Jean-Paul Latgé[2], Ping Wang[3], Tuo Wang[1*]

[1] Department of Chemistry, Michigan State University, East Lansing, MI 48824, USA

[2] Institute of Molecular Biology and Biotechnology, University of Crete, Heraklion, GR 70013, Greece

[3] Department of Microbiology, Immunology and Parasitology, Louisiana State University Health Sciences Center, New Orleans, LA 70112, USA

[†] Current Address: Renewable Resources and Enabling Sciences Center, National Renewable Energy Laboratory, Golden, CO 80401 USA

Table of Contents

Supplementary Fig. 1. Replication of *R. delemar* samples 3
Supplementary Fig. 2. Resonance assignment of chitin, chitosan, and glucan in *R. delemar* 4
Supplementary Fig. 3. Rigid cell wall polysaccharides in *R. delemar* 5
Supplementary Fig. 4. Biopolymer signals resolved in 2D ^{13}C refocused J-INADEQUATE 6
Supplementary Fig. 5. Distribution of chitin, chitosan, and β-glucan in rigid and mobile domains 7
Supplementary Fig. 6. Identification of mobile nitrogenated carbohydrates 8
Supplementary Fig. 7. Carbon connectivity of five types of fucose residues 9
Supplementary Fig. 8. Absence of galacturonic acid and glucuronic acid in *R. delemar* 10
Supplementary Fig. 9. β-glucans in the mobile domain of nikkomycin-treated *R. delemar* cell wall 11
Supplementary Fig. 10. Comparison of 1D spectra for estimation of composition 12
Supplementary Fig. 11. Cross sections of long-range correlation spectrum of apo *R. delemar* 13
Supplementary Fig. 12. Chitin is the only carbohydrate detectable in 15 ms PAR spectrum 14
Supplementary Fig. 13. Intermolecular contacts observed in nikkomycin-treated *R. delemar* 15
Supplementary Fig. 14. Proteins are partially ordered and closely packed in *R. delemar,* apo 16
Supplementary Fig. 15. Presence of protein in both mobile and rigid domains 17
Supplementary Fig. 16. Secondary structure of amino acids 18
Supplementary Fig. 17. Composition and water accessibility of proteins 19
Supplementary Fig. 18. Water-edited experiment setup for inspecting carbohydrate hydration 20
Supplementary Fig. 19. Nikkomycin alters water accessibility of rigid polysaccharides 21
Supplementary Fig. 20. ^{13}C-T$_1$ relaxation in apo and nikkomycin-treated *R. delemar* 22
Supplementary Fig. 21. ^{1}H-T$_{1\rho}$ relaxation in *R. delemar* 23
Supplementary Fig. 22. Distinct spectral patterns of *Rhizopus*, *Mucor*, and *Aspergillus* 24
Supplementary Fig. 23. Similarity of *Rhizopus* and *Mucor* carbohydrates 25
Supplementary Fig. 24. Chemical shift comparison of chitin and chitosan 26
Supplementary Fig. 25. Experimental flowchart of fungal cultivation 27
Supplementary Fig. 26. Experimental flowchart of constructing fungal growth curves 28
Supplementary Table 1. Estimation of fractions of rigid and mobile molecules 29
Supplementary Table 2. Relative abundance of rigid and mobile carbohydrates 30
Supplementary Table 3. Measurements of the cell-wall thickness of *R. delemar* 31
Supplementary Table 4. Solid-state NMR experimental parameters 32
Supplementary Table 5. ^{13}C and ^{15}N chemical shifts of rigid polysaccharides in *R. delemar* 33
Supplementary Table 6. ^{13}C chemical shifts of mobile polysaccharides in *R. delemar* 34
Supplementary Table 7. Chemical shifts of amino acids in the fungal cells 35
Supplementary Table 8. Molar composition of polysaccharides in cell walls 36
Supplementary Table 9. Intermolecular cross peaks probed by ssNMR 37
Supplementary Table 10. Water-edited intensities of polysaccharide carbon sites 38
Supplementary Table 11. ^{13}C-T$_1$ and ^{1}H-T$_{1\rho}$ relaxation time constants 39
Supplementary Table 12. ^{13}C chemical shifts of chitin and chitosan in *Rhizopus* and *Mucor* species 40
Supplementary References 41

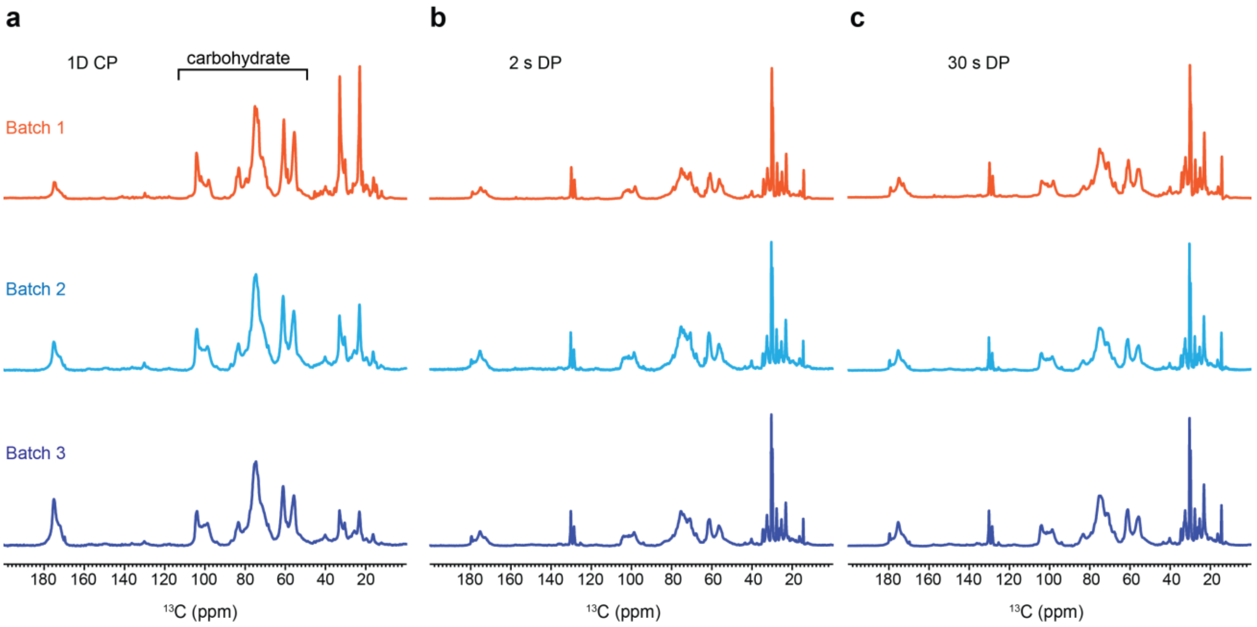

Supplementary Figure 1. Replication of *R. delemar* samples. (**a**) 1D ^{13}C CP spectra of apo *R. delemar*. (**b**) Mobile components detected by 1D ^{13}C DP with a short recycle delay of 2 s. (**c**) Quantitative ^{13}C DP spectra for the detection of all molecules through a recycle delay of 30 s. The spectra exhibit high reproducibility across three separate batches prepared in 2018 (batch 1), 2022 (batch 2) and 2023 (batch 3).

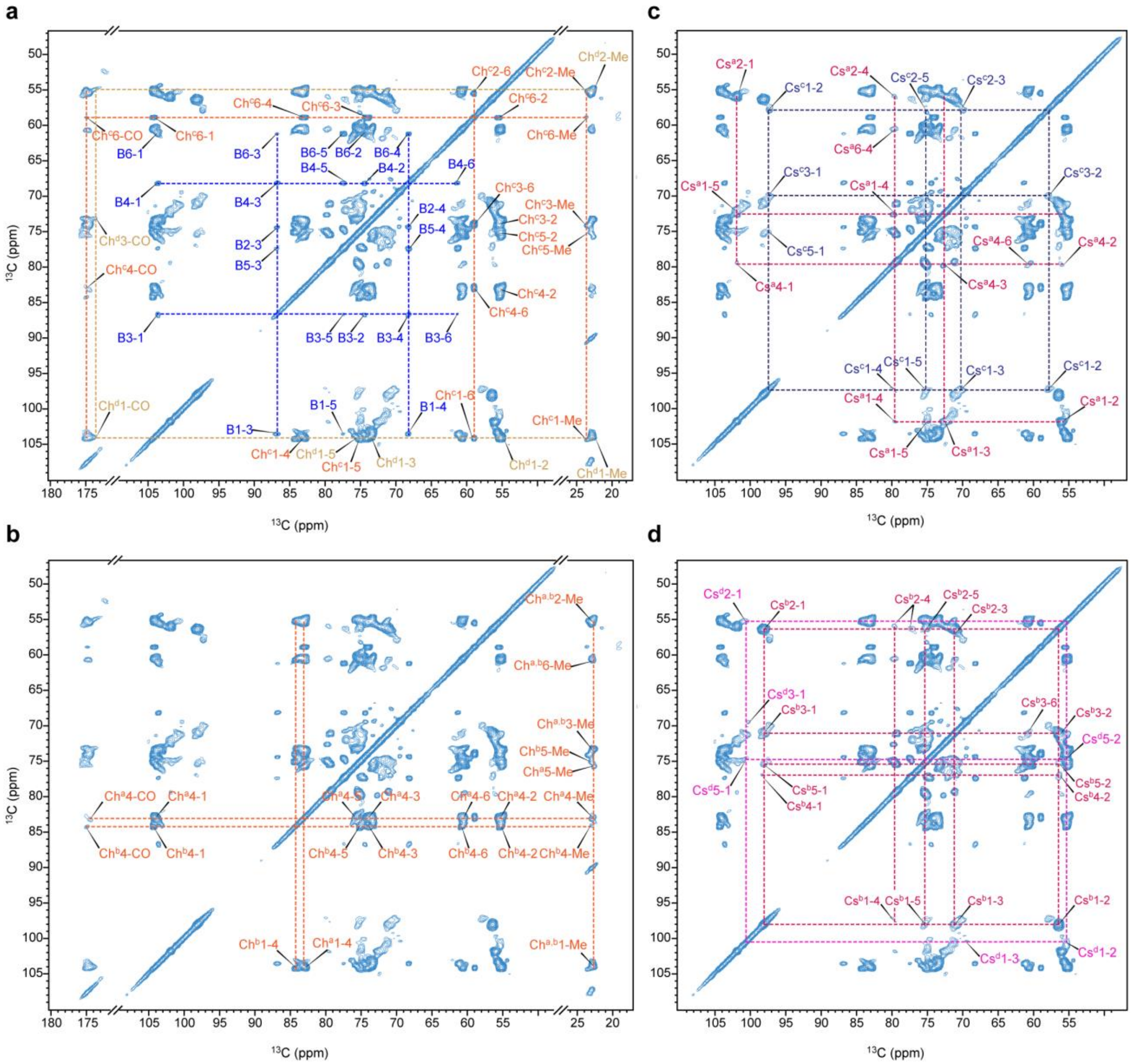

Supplementary Figure 2. Resonance assignment of chitin, chitosan, and glucan in *R. delemar* cell wall. CP-based 2D ^{13}C-^{13}C correlation spectrum measured with 53 ms CORD mixing were plotted as four separate panels to show the well-resolved signals of **(a)** β-1,3-glucan and type-c and type-d of chitin forms, **(b)** type-a and type-b chitin, **(c)** type-a and type-c chitosan molecules, and **(d)** type-b and type-d chitosan molecules. NMR abbreviations are used for β-1,3-glucan (B), chitin (Ch), and chitosan (Cs). Superscripts indicate the subform type within each molecule. Carbon numbers are also labeled for each cross peak. For example, Ch^{c}2-Me indicates the cross peak between carbon 2 and methyl carbon within type-c chitin.

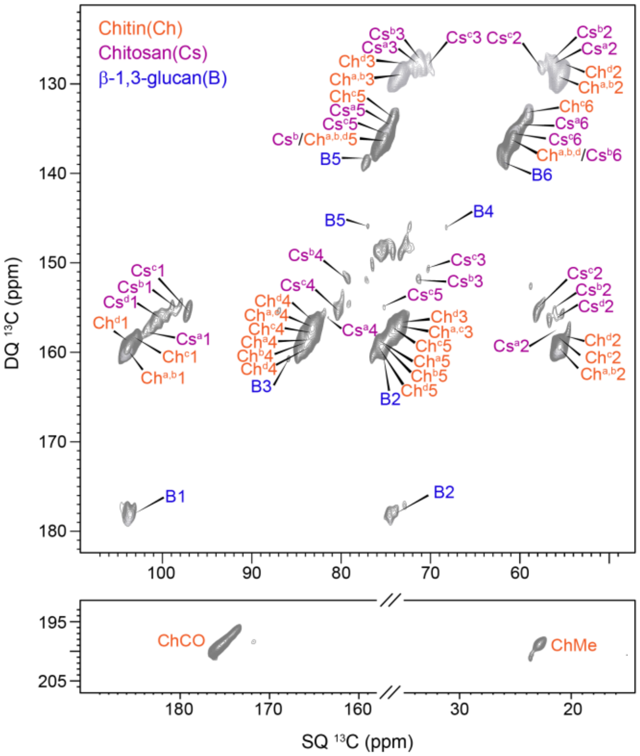

Supplementary Figure 3. Rigid cell wall polysaccharides in *R. delemar*. Through-bond carbon connectivity was resolved using 2D ^{13}C CP refocused J-INADEQUATE spectrum. Multiple sets of conformers were resolved for chitin and chitosan, in addition to a single type of β-1,3-glucan. Each peak is annotated with the abbreviation of the carbohydrate name, the subtype (in superscript), and the carbon number. For instance, Ch^c5 represents the carbon 5 of type-c chitin. The linkage between the carbonyl (CO) and methyl (Me) of chitin is shown in the bottom panel.

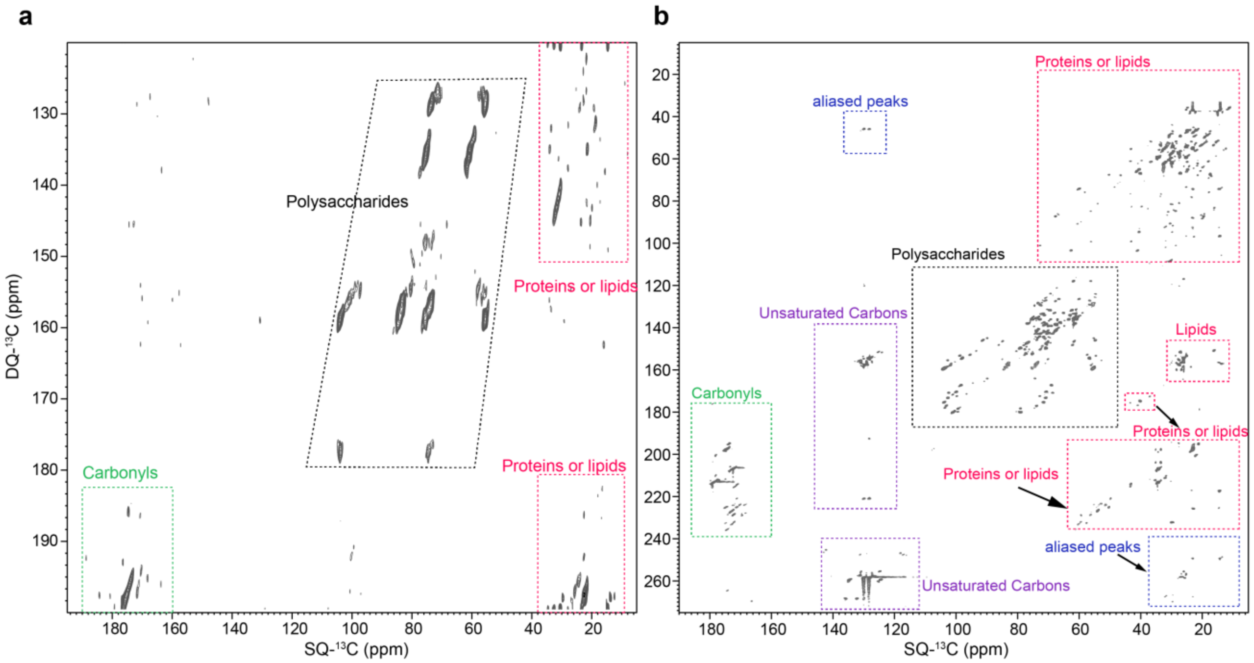

Supplementary Figure 4. Biopolymer signals resolved in 2D ^{13}C refocused J-INADEQUATE. (**a**) Rigid components of the cell wall in apo *R. delemar* sample probed using 2D CP ^{13}C refocused J-INADEQUAT E spectrum. The individual regions were marked with different colors of quadrangles: green for carbonyls, black for polysaccharides, and red for aliphatics from proteins and lipids. (**b)** Mobile components of apo *R. delemar* sample detected using 2D DP ^{13}C refocused J-INADEQUATE spectrum. The individual regions are marked using the same color code mentioned above. The unsaturated carbons (aromatics or C=C) are marked in purple and the aliased peaks are boxed in blue.

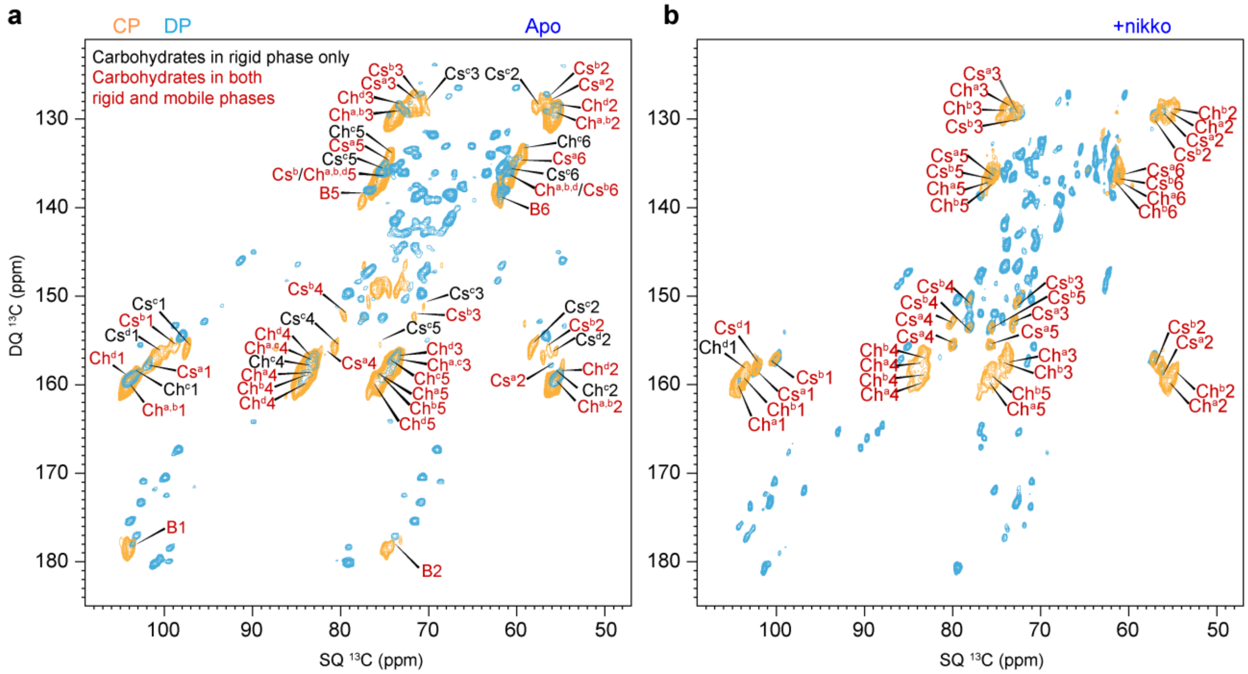

Supplementary Figure 5. Distribution of chitin, chitosan, and β-glucan in rigid and mobile domains. Overlay of 2D refocused J-INADEQUATE spectra measured with CP (orange) and DP (cyan) for (**a**) apo and (**b**) nikkomycin-treated *R. delemar* samples. The carbohydrates observed only in the CP-based spectra are rigid and are marked in black. The carbohydrates observed in both CP (INADEQUATE here and CORD in Figure S2) and DP-based spectra have two-modal distribution in rigid and mo bile phases, and are marked in red. Types-a,b chitin, types-a,b chitosan and β-1,3-glucan were observed in both domains for both samples. The Peak (DQ~159.8 ppm, SQ~104.1 ppm) of the ^{13}C DP J-INADEQUATE spectra (blue) in panel b are assigned as types-a,b chitin, but other carbons of them (C3, C4, C5, and C6) are not confirmed.

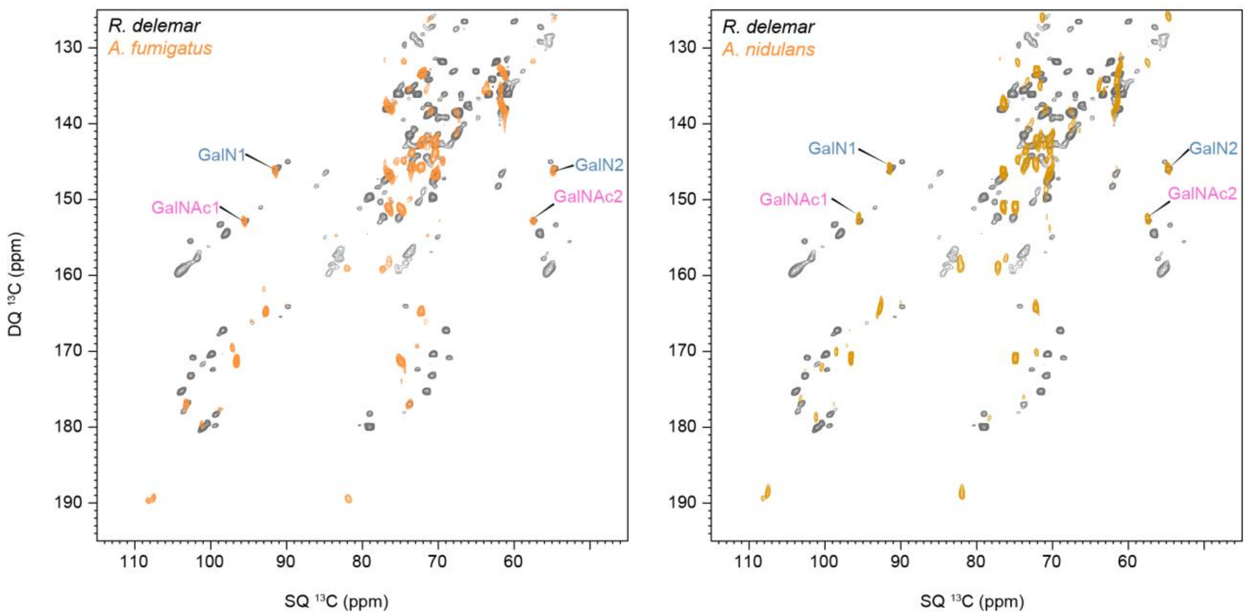

Supplementary Figure 6. Identification of mobile nitrogenated carbohydrates. One type of GalNAc and GalN are respectively confirmed by overlapping the 2D DP ^{13}C J-INADEQUATE spectrum of *R. delemar* with two *Aspergillus* species (*A. fumigatus* and *A. nidulans*). The signals of galactosamine (GalN) and N-acetylgalactosamine (GalNAc) present in the mobile region of *R. delemar* are well-matched with the two A*spergillus* samples.

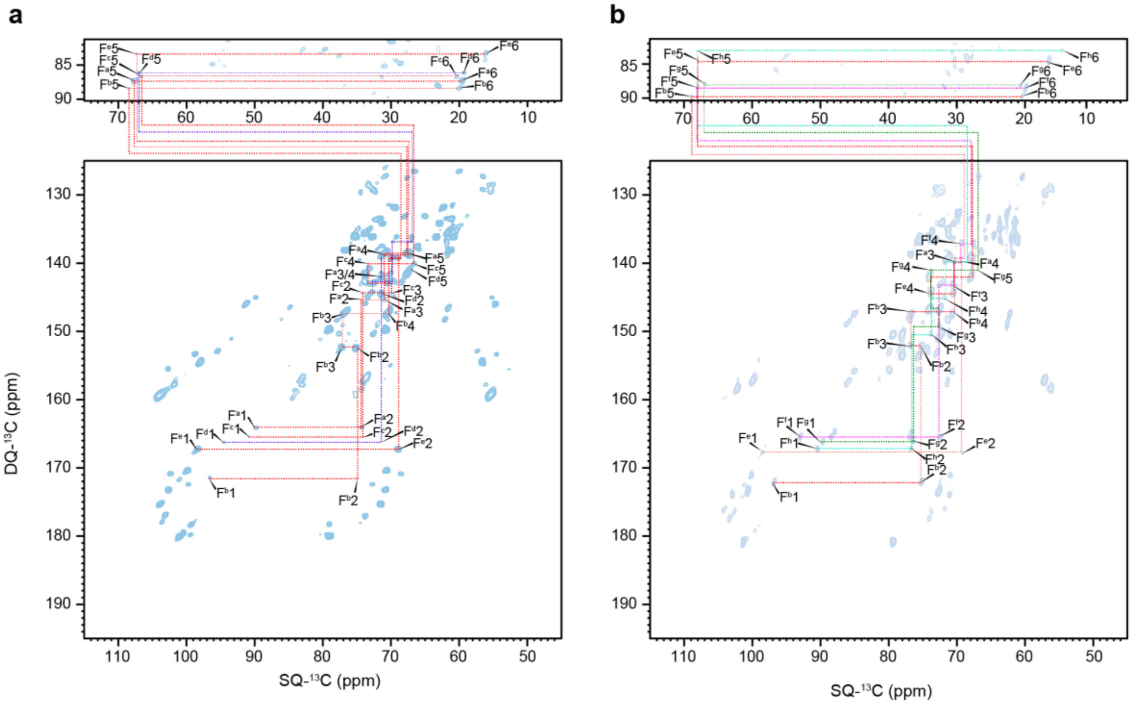

Supplementary Figure 7. Carbon connectivity of five types of fucose residues. The connectivity is tracked starting from the unique C5-C6 pair that involves a methyl carbon and a carbon on the pyranose ring of the carbohydrate. Similar types of fucose residues can be found in the ^{13}C DP refocused INADEQUATE spectra of both (**a**) apo and (**b**) nikkomycin-treated *R. delemar* samples.

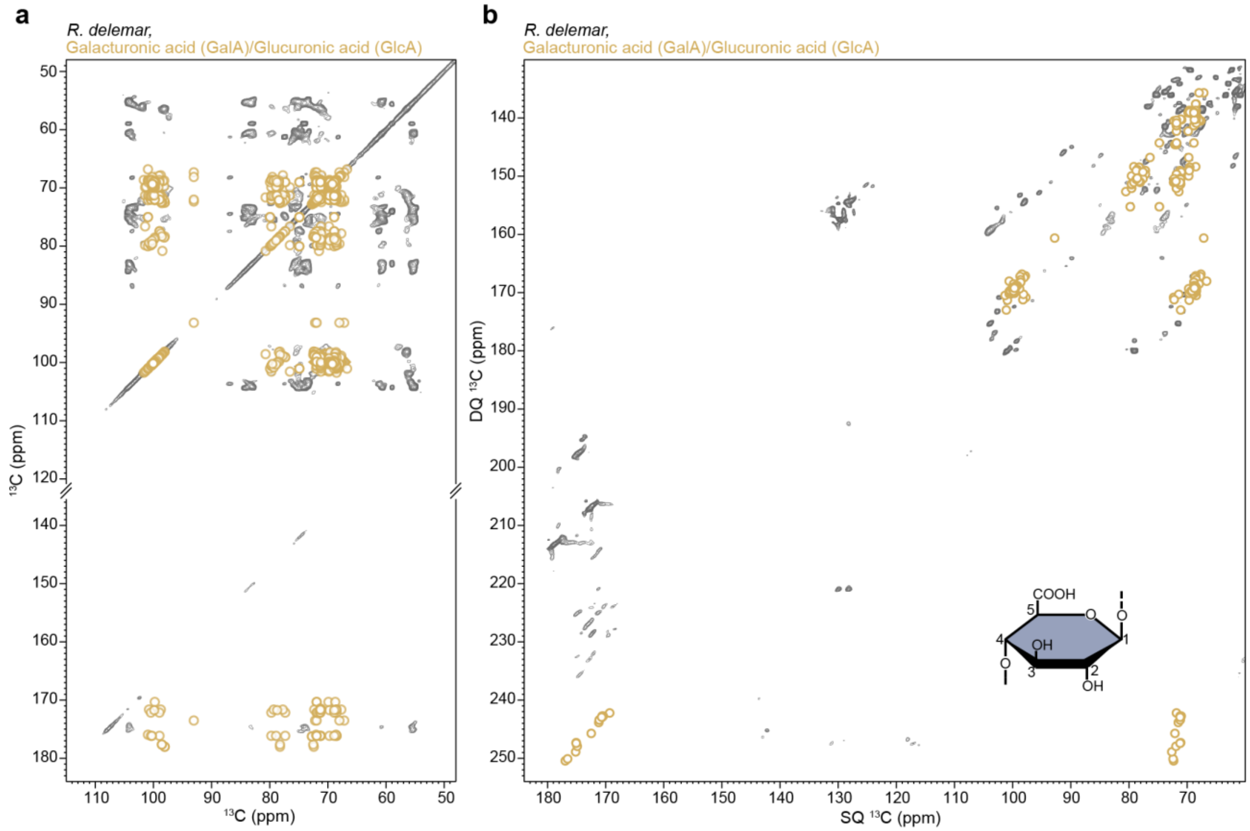

Supplementary Figure 8. Absence of galacturonic acid and glucuronic acid in *R. delemar* samples. Overlay of experimentally measured spectra (plotted in grey) with simulated 2D spectra (plotted in yellow) constructed using CCMRD-deposited chemical shifts of galacturonic acid or glucuronic acid. Galacturonic acid and glucuronic acid are not observed within (**a**) 53-ms ^{13}C-^{13}C CORD spectrum or (**b**) ^{13}C DP J-INADEQUATE; therefore, they are not present in either the rigid or the mobile portions of the cell wall. A representative structure of an GlcA residue is shown.

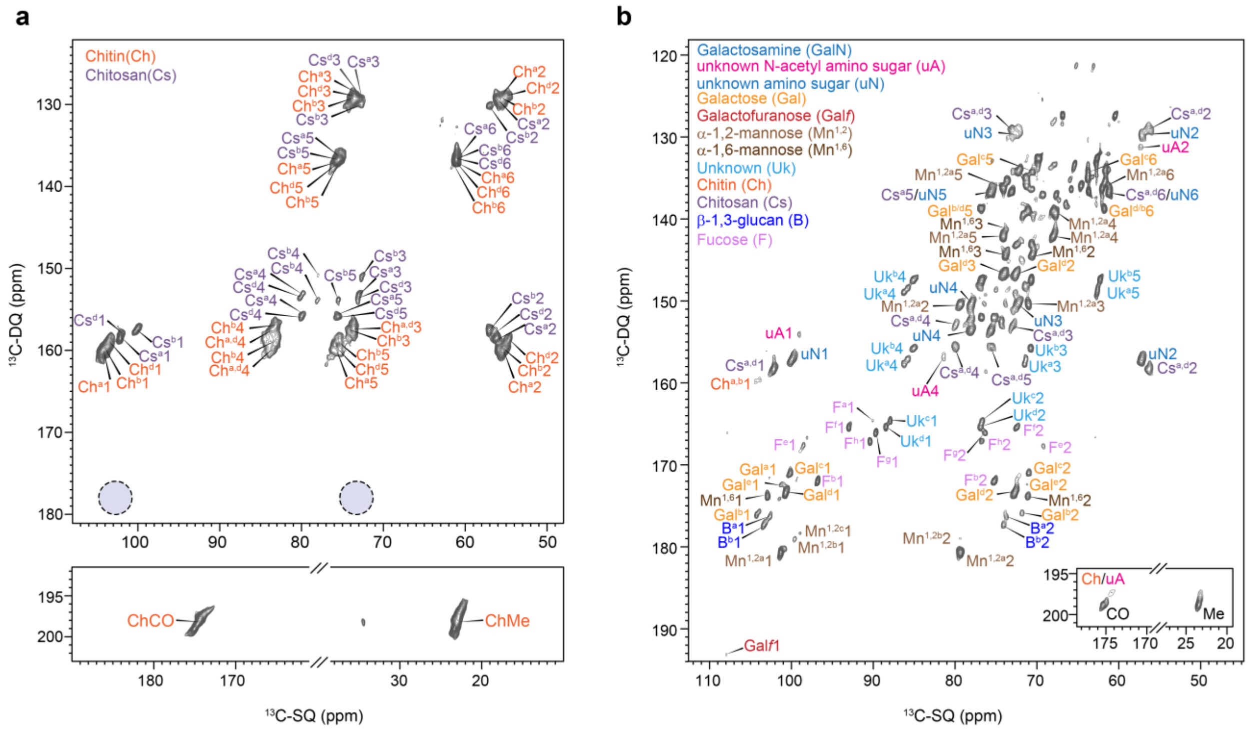

Supplementary Figure 9. β-glucans in the mobile domain of nikkomycin-treated *R. delemar* cell walls. (**a**) CP-based 2D ^{13}C refocused J-INADEQUATE spectrum detecting rigid molecules. β-glucan is absent as highlighted by the dashed line circles. (**b**) Mobile carbohydrates detected by DP-based 2D ^{13}C refocused J-INADEQUATE spectrum, with signals from β-1,3-glucan (abbreviated as B). Each peak is annotated with the abbreviation of the carbohydrate name, the subtype (in superscript), and the carbon number.

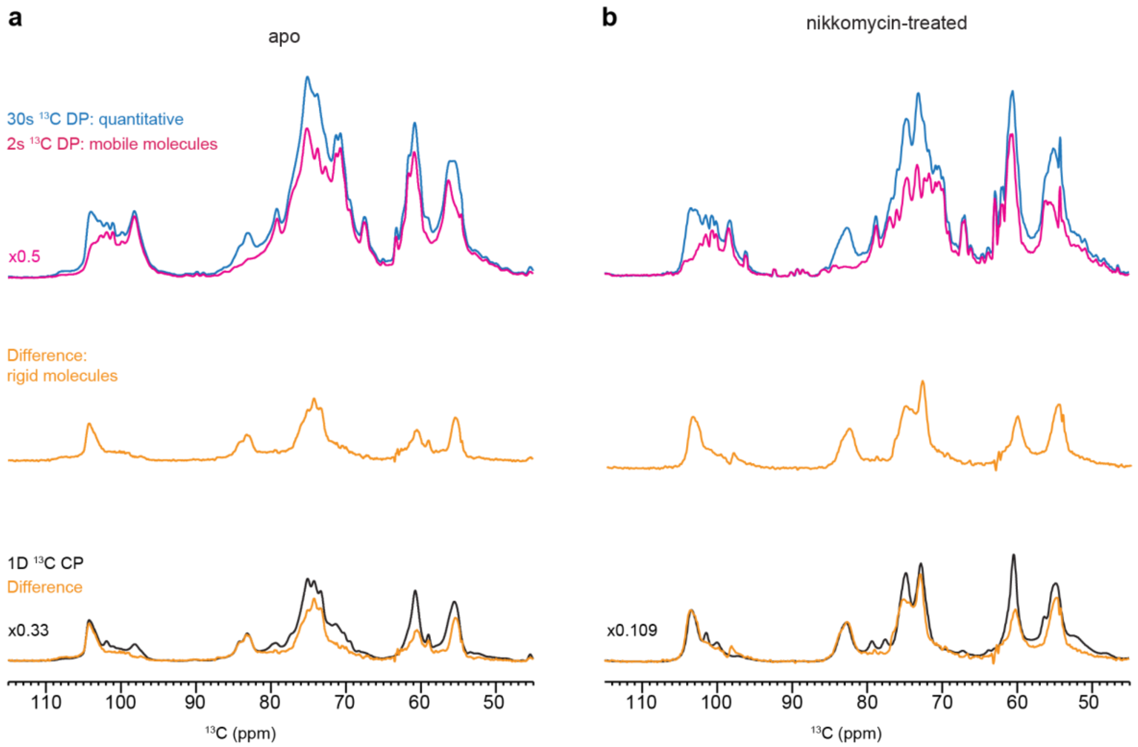

Supplementary Figure 10. Comparison of 1D spectra for estimating mobile and rigid fractions. 1D ^{13}C spectra are compared for (**a**) apo *R. delemar* and (**b**) nikkomycin-treated *R. delemar*. The top row shows the overlay of ^{13}C DP spectra measured with recycle delays of 30 s (cyan) and 2 s (magenta) for detecting all molecules and mobile molecules, respectively. The 2s DP spectrum of the apo sample is scaled down by half due to having twice the number of scans compared to the 30-second DP spectrum of the same sample. The second row presents the difference spectra, which are generated by subtracting the 2-second DP spectrum from the 30-second DP spectrum, thereby emphasizing rigid molecules. The bottom row shows an overlay of this difference spectrum with the ^{13}C cross polarization (CP) spectrum. The CP spectra are scaled (with the scaling factors indicated) such that the peak intensities of the difference spectrum do not surpass those of the ^{13}C CP, considering that the latter might also detect signals from semi-mobile molecules. The estimated carbon percentages of mobile and rigid carbohydrates are detailed in **Supplementary Tables 1** and **2**.

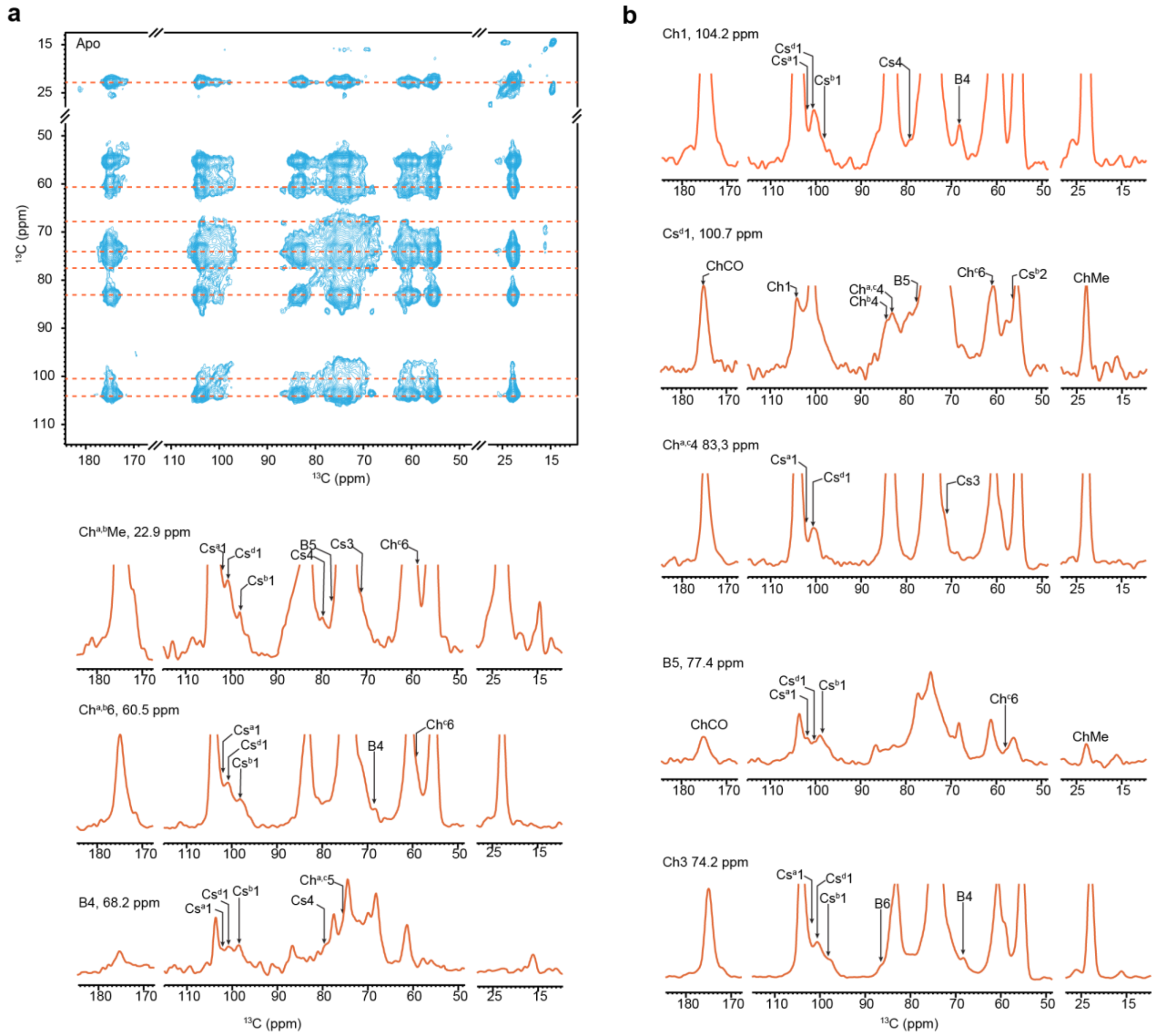

Supplementary Figure 11. Cross sections of long-range correlation spectrum of apo *R. delemar*. (a) 2D ^{13}C 1-s PDSD spectrum of apo *R. delemar* cell wall. Dashed lines indicate the positions where 1D cross sections are extracted. (**b**) 1D cross sections extracted from 1s PDSD spectrum. The ω_1 chemical shift and assignment are shown for each cross section. Key intermolecular cross peaks are also labeled.

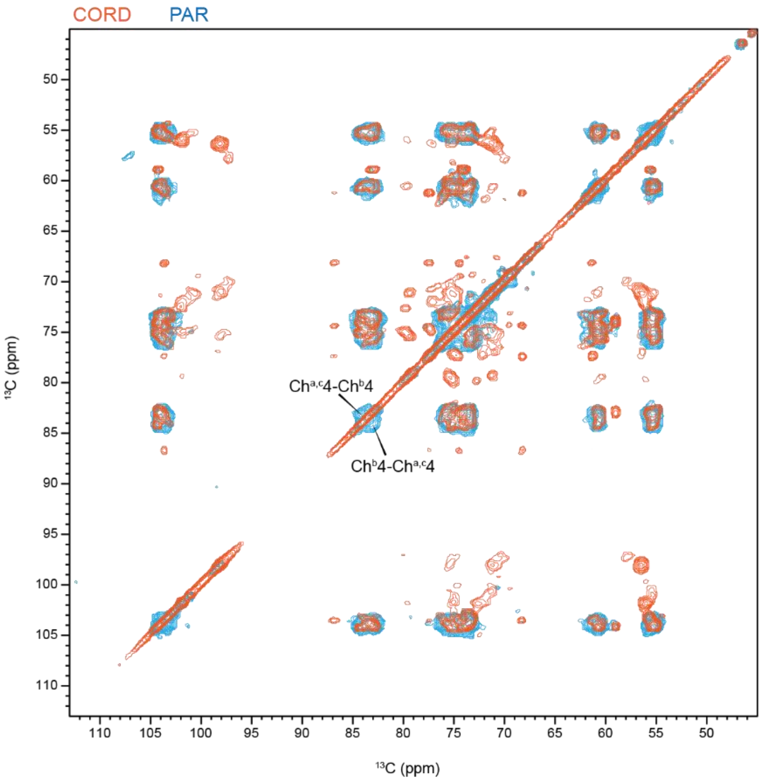

Supplementary Figure 12. Chitin is the only carbohydrate detectable in 15 ms PAR spectrum. The overlay of 53 ms CORD spectrum (orange) and 15 ms PAR spectrum (cyan) of *R. delemar*, apo revealed that only chitin signals are retained in both spectra; therefore, this fraction of chitin signals are highly crystalline. The signals from β-glucan and chitosan disappeared in PAR, revealing the disordered nature of these two types of molecules though they are still physically associated with chitin domains. Two cross peaks are observed between different types of chitin molecules in the PAR spectra.

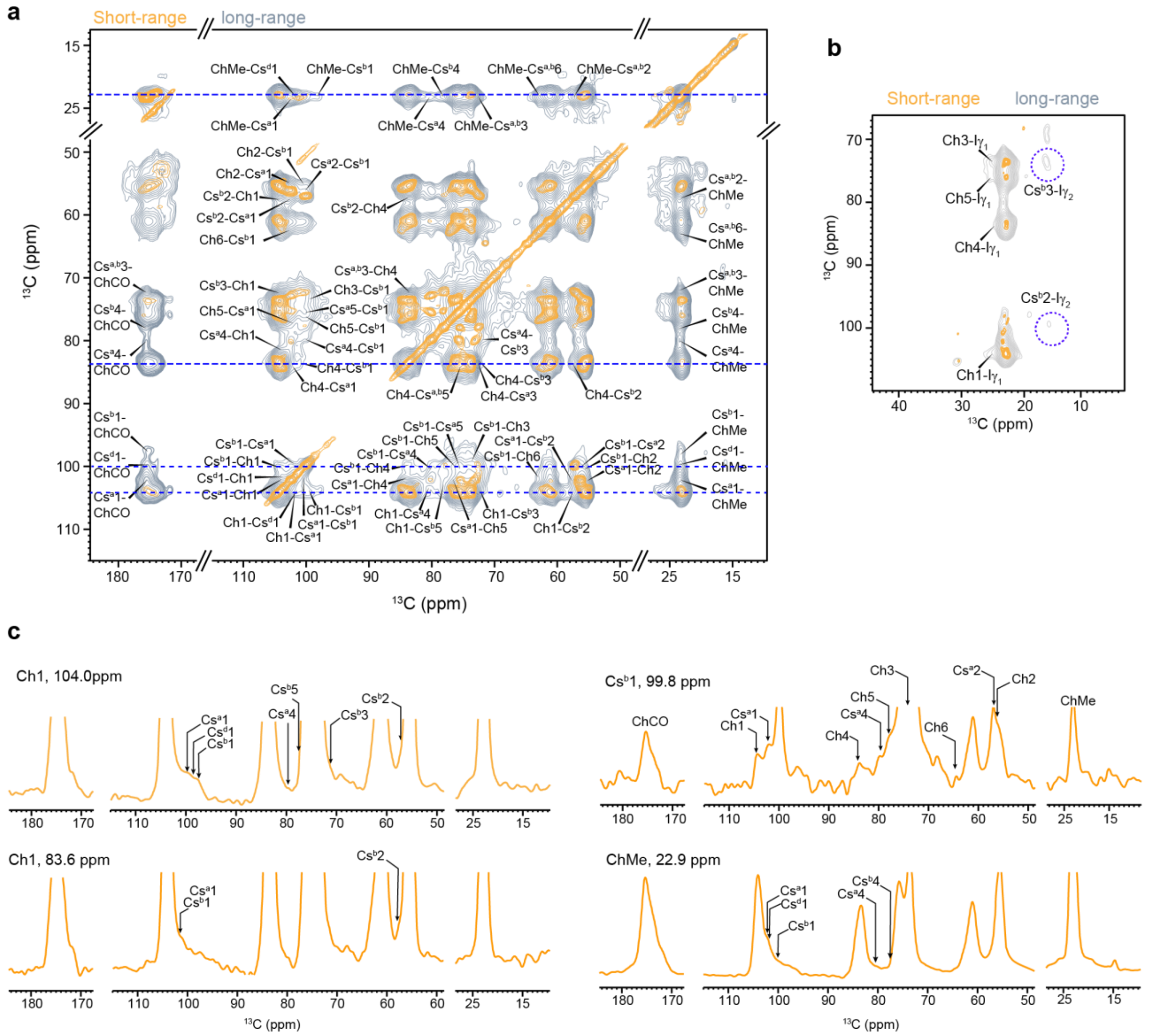

Supplementary Figure 13. Intermolecular contacts observed in nikkomycin-treated *R. delemar*. (**a**) Overlay of 2D ^{13}C-^{13}C 1-s PDSD (grey) with 2D 53-ms CORD spectra (yellow) measured on nikkomycin-treated *R. delemar* cells an 800 MHz spectrometer at 273 K (1s PDSD) or 290 K (53ms CORD) under 15 kHz MAS. Intermolecular cross peaks among rigid polysaccharides are labeled. Dashed lines indicate the positions where 1D cross sections are extracted. (**b**) Spectral region (CORD in yellow and PDSD in grey) showing spatial contacts between protein and polysaccharides where the corresponding cross peaks are identified for chitin-isoleucine and chitosan-isoleucine. (**c**) Representative cross sections extracted from 2D 1-s PDSD spectrum.

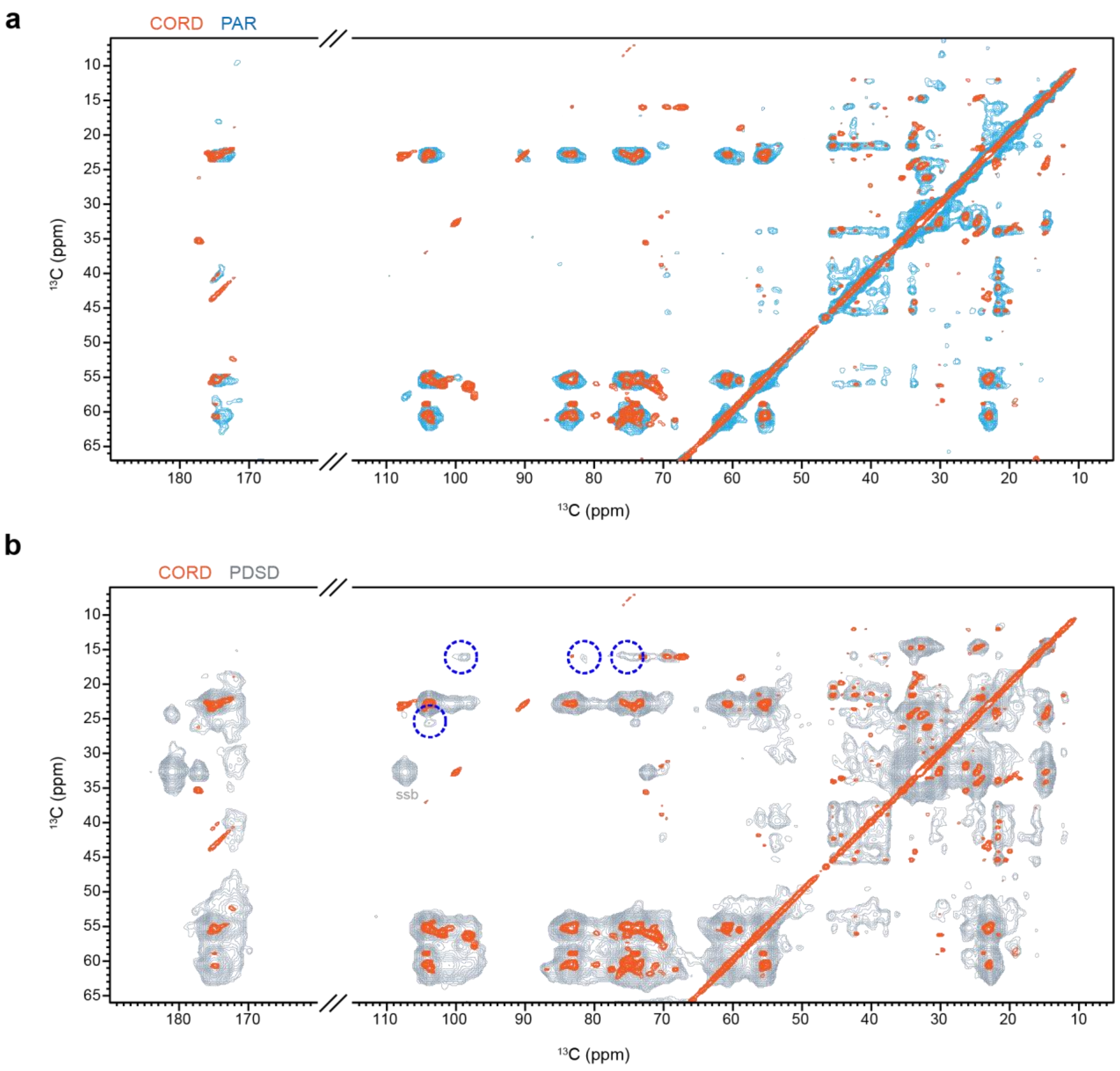

Supplementary Figure 14. Proteins are partially ordered and closely packed in *R. delemar*, apo. (a) Overlay of 2D ^{13}C-^{13}C correlation spectra measured with 53 ms CORD (orange) and 15 ms PAR (cyan). **(b)** Overlay of 2D spectra measured with 53 ms CORD (orange) and 1 s PDSD (grey). Proteins showed largely equilibrated signals in both PAR and PDSD spectra.

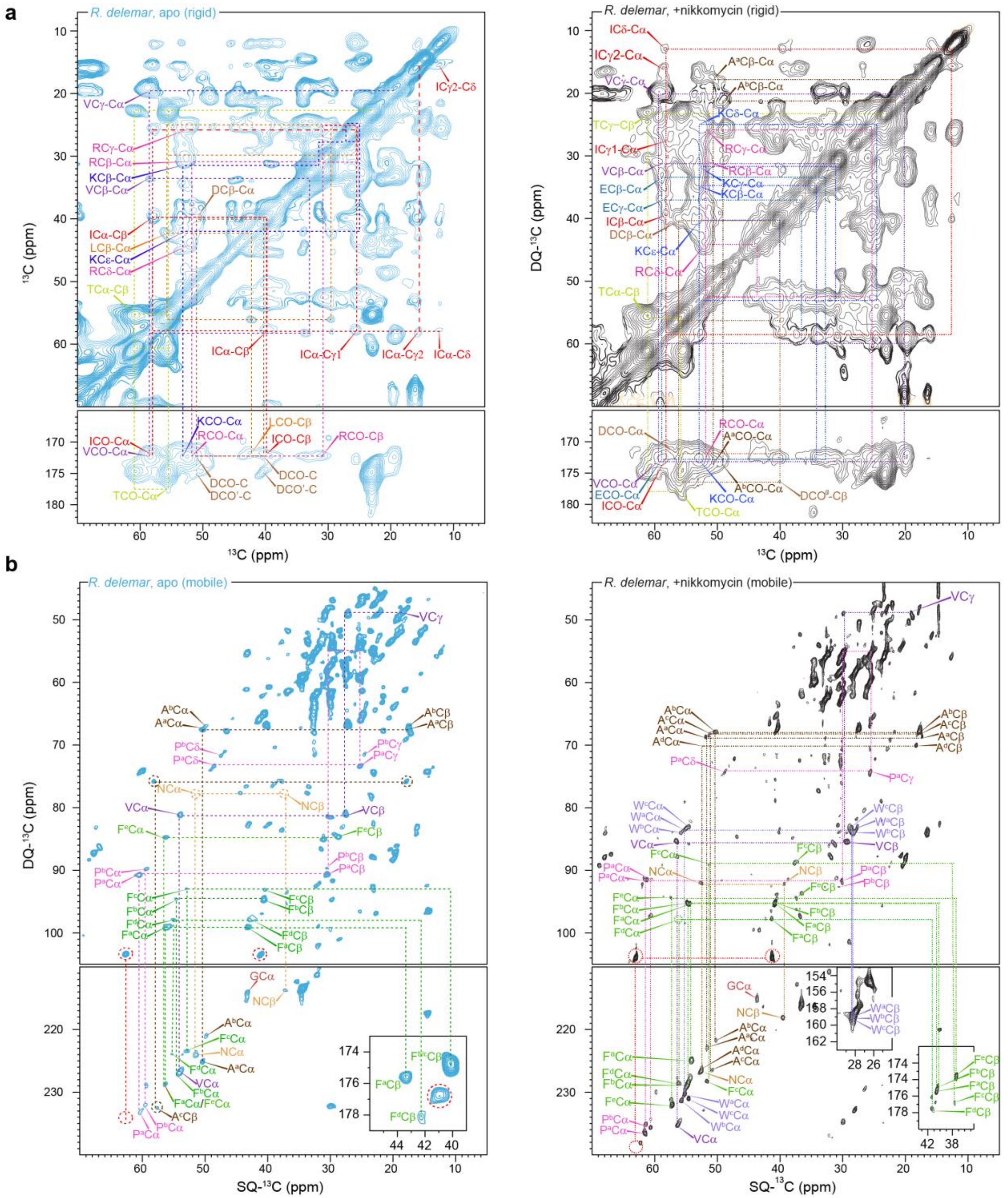

Supplementary Figure 15. Presence of protein in both mobile and rigid domains. (**a**) 2D ^{13}C-^{13}C CP-based 53 ms CORD spectra of *R. delemar* samples (left, apo; right, nikkomycin-treated) showing signals of proteins in the rigid domains. Both spectra were measured on an 800 MHz spectrometer and were processed using Gaussian multiplication (GM) with LB of -30 Hz and GB of 0.03 for both dimensions (F1 and F2). This processing enhanced sensitivity to reveal otherwise weak protein signals. (**b**) 2D ^{13}C DP refocused J-INADEQUATE spectra detecting mobile proteins. Single-letter abbreviations are used for the identified amino acids.

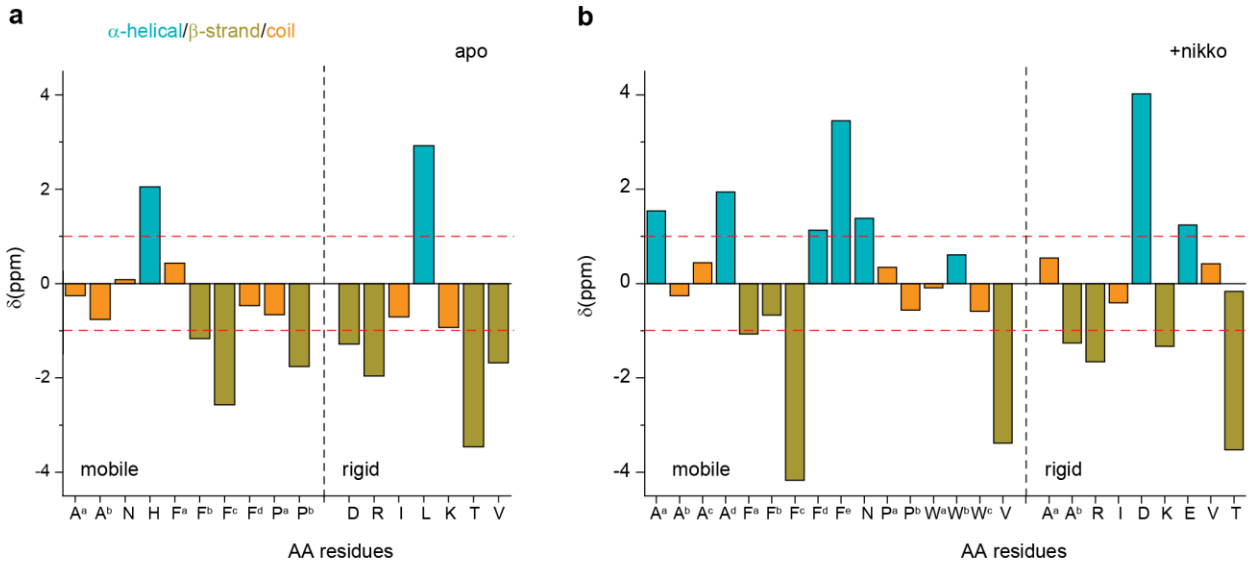

Supplementary Figure 16. Secondary structural conformations predictions of amino acid residues. *R. delemar*, apo (left panel) and *R. delemar*, + nikko (right panel), the amino acids were identified using ^{13}C DP refocused J-inadequate (mobile amino acids), and CP-based 2D ^{13}C-^{13}C 53 ms-CORD to probe the amino acids in rigid domain. The determination of protein secondary structure relies on the ^{13}C chemical shifts of Cα and Cβ by using equation: $\boldsymbol{\delta}\ (\mathbf{ppm}) = \left(\boldsymbol{C_{\alpha}^{Sample}} - \boldsymbol{C_{\alpha,coil}^{reference}}\right)$. The α-helical, random coil and β-strand conformations are depicted in light steel blue, grey and pale turquoise, respectively. Red dashed lines represent errors (full width at half maximum estimated from the corresponding peaks) in chemical shifts. Within this error range, amino acids are considered to have random coil conformations. Amino acids in the mobile domain (left) and rigid domain (right) are separated by black dashed lines. Source data are provided in a Source Data file.

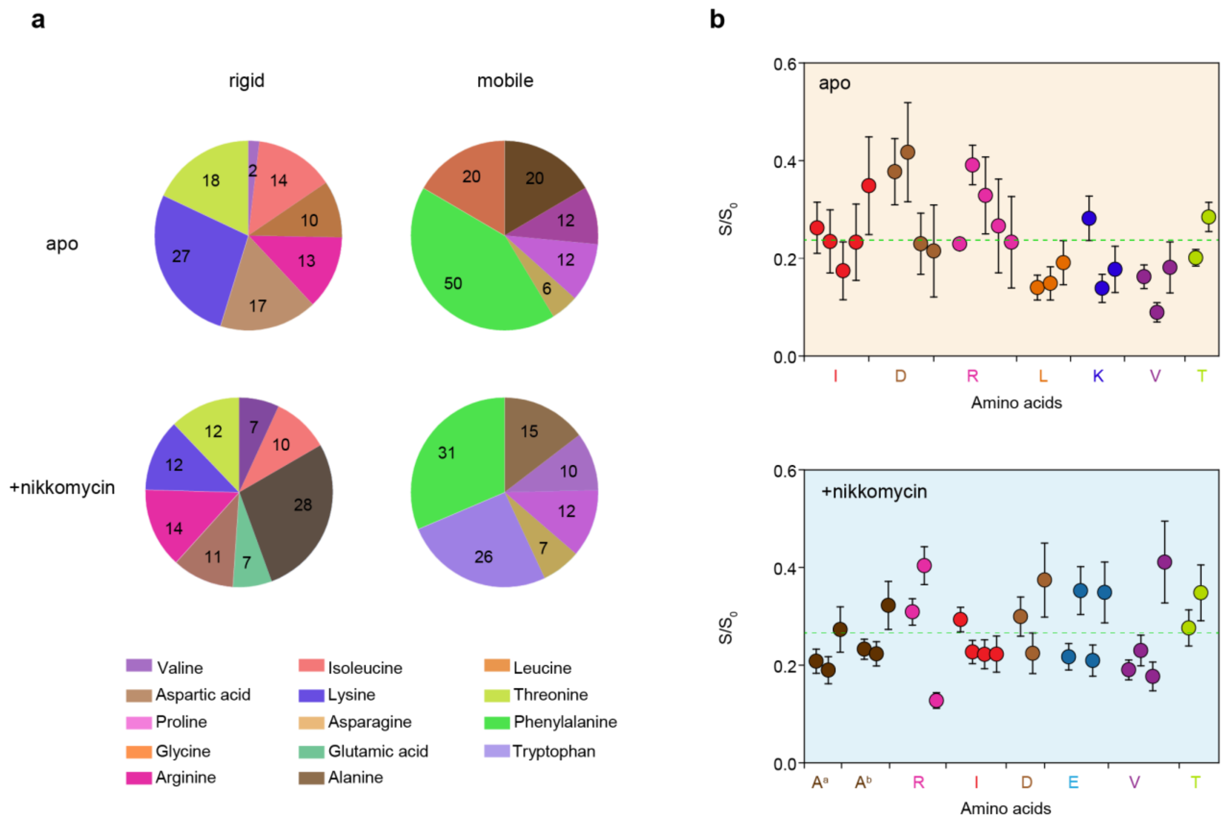

Supplementary Figure 17. Composition and water accessibility of proteins in *Rhizopus* samples. (**a**) Relative abundances of identified amino acid residues in the rigid and mobile domains. The composition is estimated using the integrals of peaks identified in 2D spectra. Different amino acid types are color-coded. (**b**) Water-edited intensities (S/S_0) of different amino acid residues. Error bars represent standard deviations propagated from NMR sensitivity. Green dashed lines indicate the average S/S_0 for all identified amino acids: 0.24 for the apo sample and 0.27 for the nikkomycin-treated sample. Source data are provided in a Source Data file.

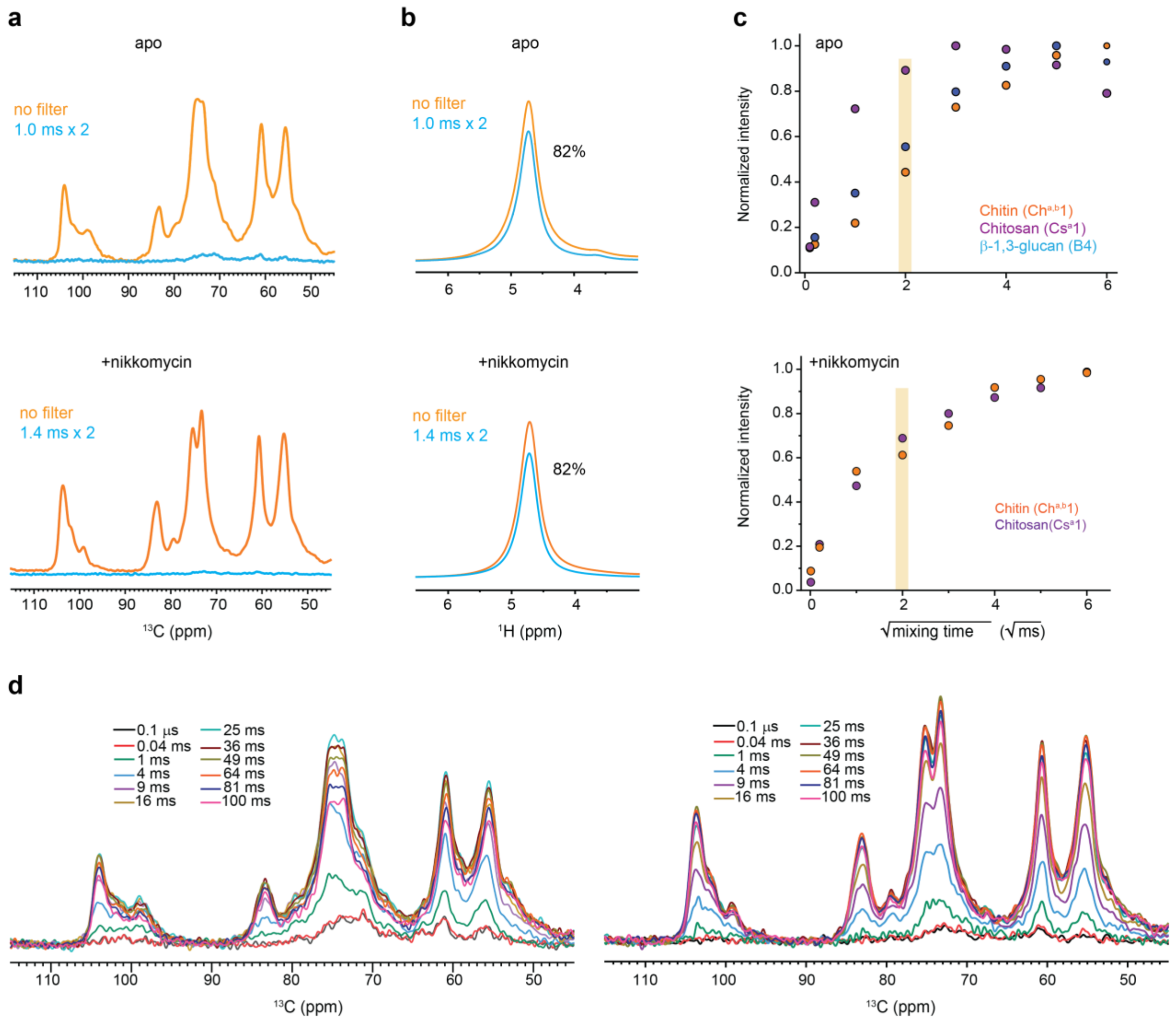

Supplementary Figure 18. Water-edited experiment setup for inspecting carbohydrate hydration. (**a**) ^{1}H-T_2 filtered (blue) and control (orange) ^{13}C spectra are shown for apo (top) and nikkomycin-treated (bottom) *R. delemar* samples. No spin diffusion was applied. Approximately 95% of carbohydrate ^{13}C signals were removed by the T_2 filter. (**b**) ^{1}H-T_2 filtered (blue) and control (orange) ^{1}H NMR spectra, with 82% of water signal retained for both samples after the ^{1}H T_2 filter. (**c**) Representative water-to-polysaccharide ^{1}H spin diffusion buildup curves. (**d**) 1D water-edited ^{13}C spectra with different ^{1}H mixing times are shown for apo and nikkomycin-treated *R. delemar* samples. All spectra were measured on 400 MHz spectrometer at 15 kHz MAS at 280 K.

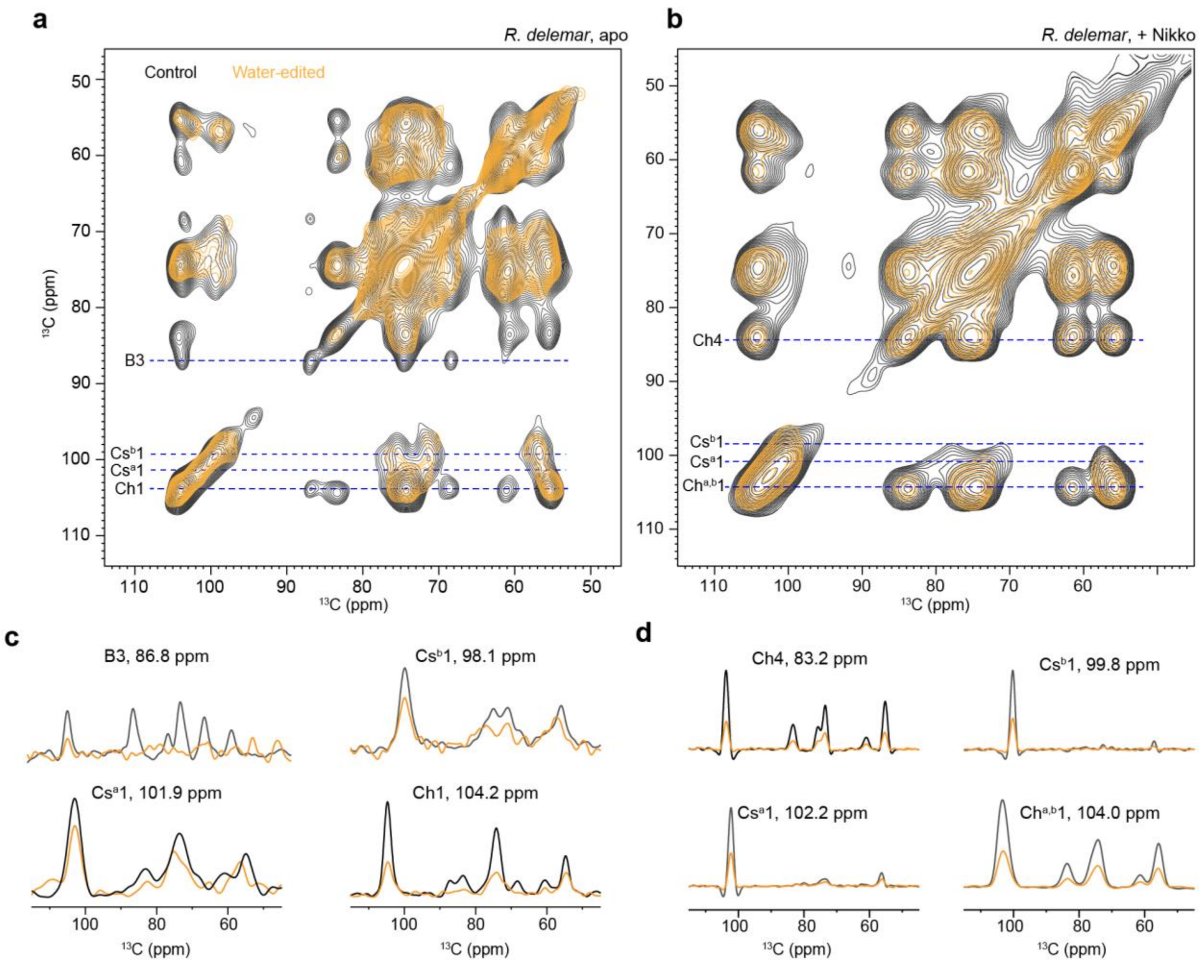

Supplementary Figure 19. Nikkomycin alters water accessibility of rigid polysaccharides. (**a**) and (**b**) Overlay of 2D water-edited (orange) and control (black) ^{13}C-^{13}C correlation spectra (T_2 = 1.0 ms x 2 for apo and T_2=1.4 ms × 2 for nikkomycin-treated *R. delemar* and τ_{SD} = 4 ms) for both *R. delemar* and nikkomycin-treated *R. delemar*. The spectra were collected on a 400 MHz NMR spectrometer under 15 kHz MAS at 280 K. Representative 1D slices extracted from the 2D ^{13}C-^{13}C correlation spectra are shown for (**c**) apo *R. delemar* sample and (**d**) nikkomycin-treated *R. delemar* sample. The control data are displayed as black solid lines, and the water-edited spectra are plotted in orange. All spectra were processed with Gaussian multiplication window function with -30 Hz LB and 0.03 GB (applied in both F1 and F2 dimensions).

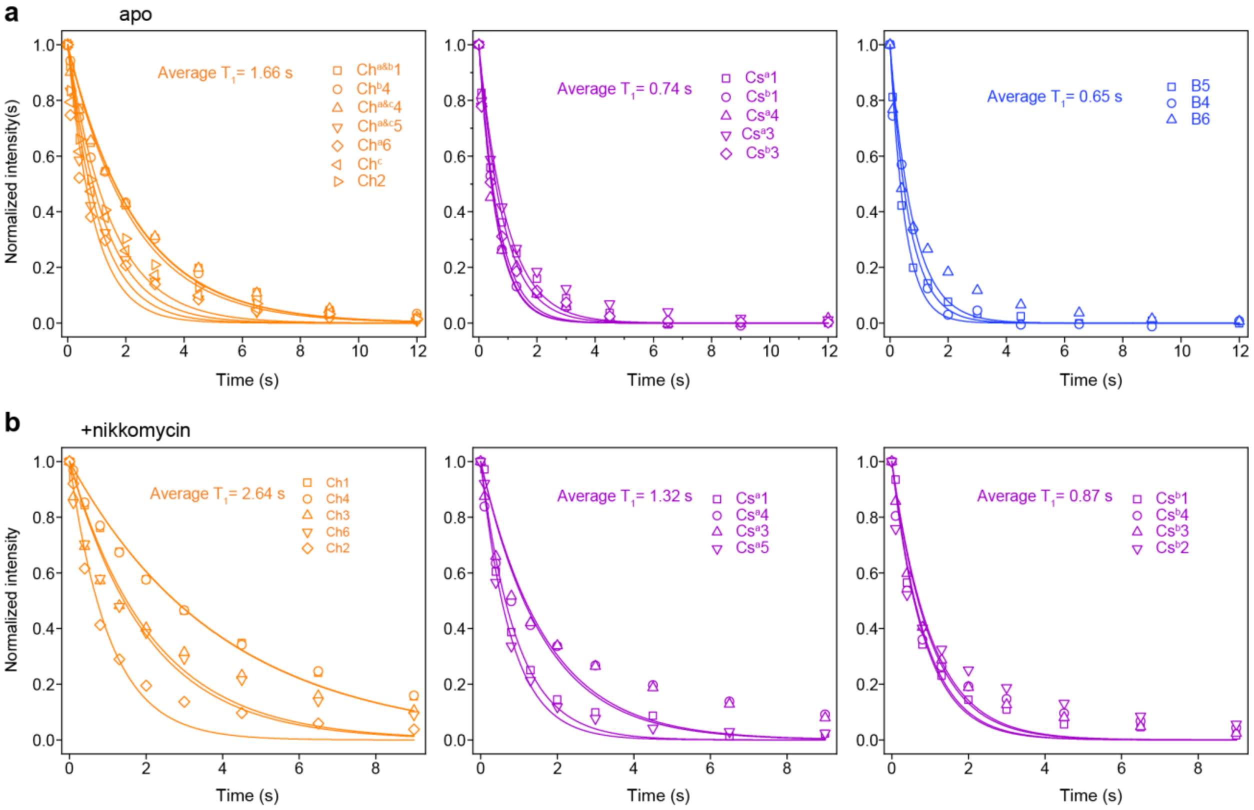

Supplementary Figure 20. ^{13}C-T$_1$ of polysaccharides in apo and nikkomycin-treated *R. delemar*. ^{13}C-T$_1$ measured with Torchia CP for (**a**) apo and (**b**) nikkomycin-treated samples. The data are separately presented for chitin (orange), chitosan (purple), and β-1,3-glucan (blue). The acquired data were fitted to a single exponential decay equation. Different symbols and color codes are used to represent different carbons in these polysaccharides.

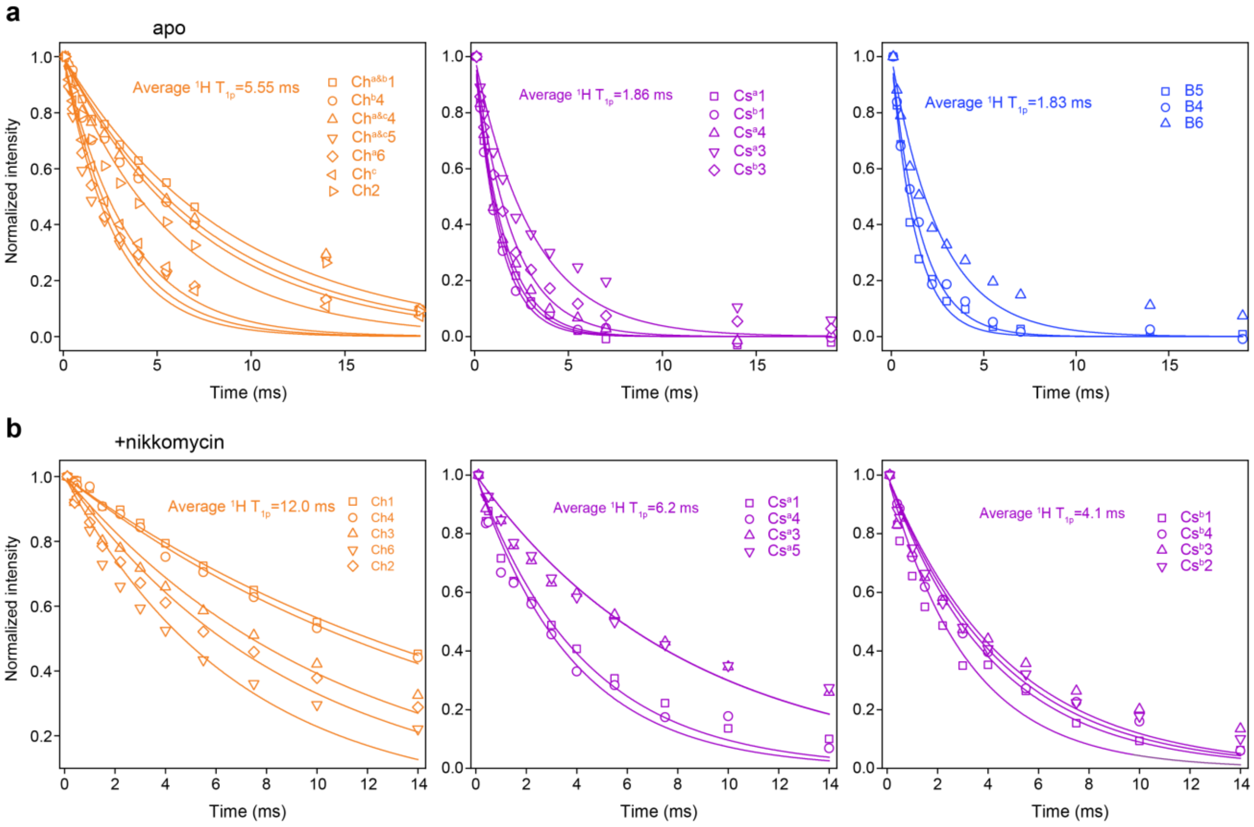

Supplementary Figure 21. ^{1}H-T$_{1\rho}$ of polysaccharides in *R. delemar* samples. ^{1}H-T$_{1\rho}$ relaxation curves of (**a**) apo and (**b**) nikkomycin-treated *R. delemar* cell walls. The data are separately presented for chitin (orange), chitosan (purple), and β-1,3-glucan (blue). The symbolic representation of each resolvable carbon site is provided.

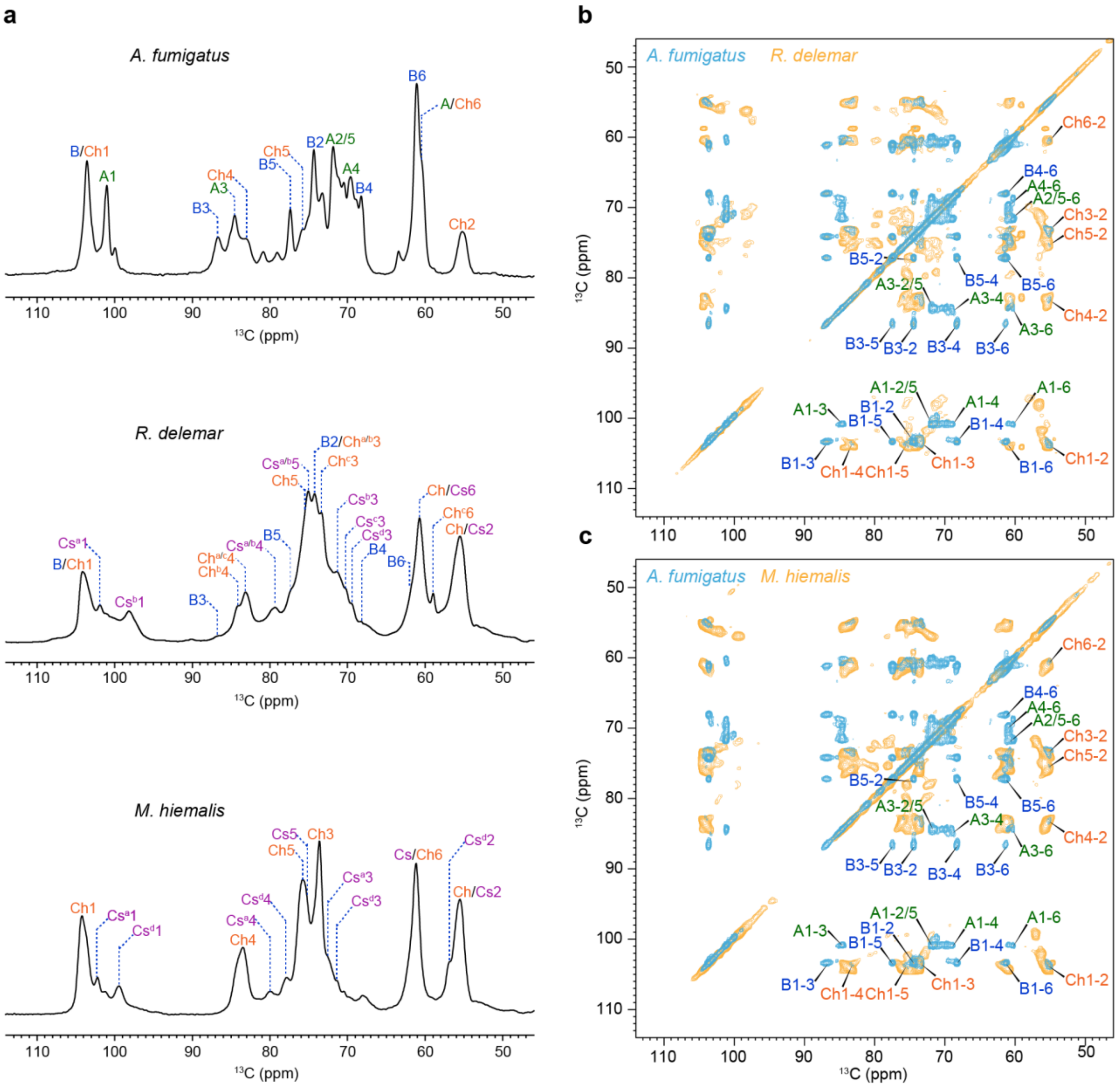

Supplementary Figure 22. Distinct spectral patterns of *Rhizopus*, *Mucor*, and *Aspergillus* species. (**a**) Comparison of 1D ^{13}C CP spectra for *A. fumigatus* (top), *R. delemar* (middle), and *M. hiemalis* (bottom). (**b**) Overlay of 2D ^{13}C CORD spectra measured on *A. fumigatus* (cyan) and *R. delemar* (orange). (**c**) Overlay of 2D ^{13}C CORD spectra measured on *A. fumigatus* (cyan) and *M. hiemalis* (orange). The spectra of *A. fumigatus* were adapted from Chakraborty et al.,[1] which is an open access publication. Resonance assignments of *A. fumigatus* polysaccharides were shown for 2D spectra. All spectra were collected on 800 MHz NMR spectrometers.

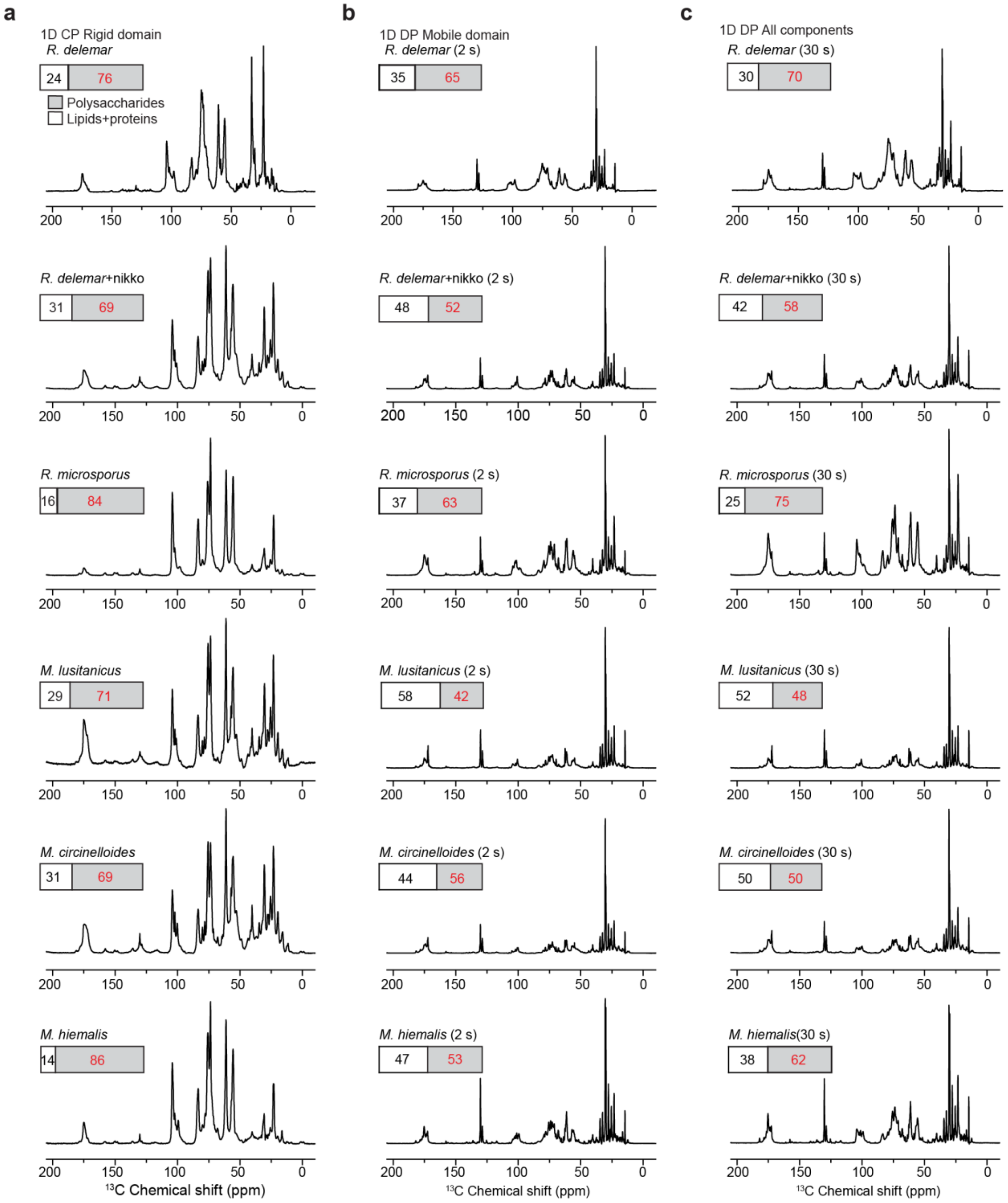

Supplementary Figure 23. Similarity of *Rhizopus* and *Mucor* carbohydrates. (**a**) 1D ^{13}C CP spectra for the detection of the rigid components within fungal cells. (**b**) and (**c**) 1D ^{13}C DP spectra measured with both short recycle delays of 2 s (mainly detects mobile components) and long recycle delays of 30 s (enabling quantitative detection for all components of the intact fungal cells).

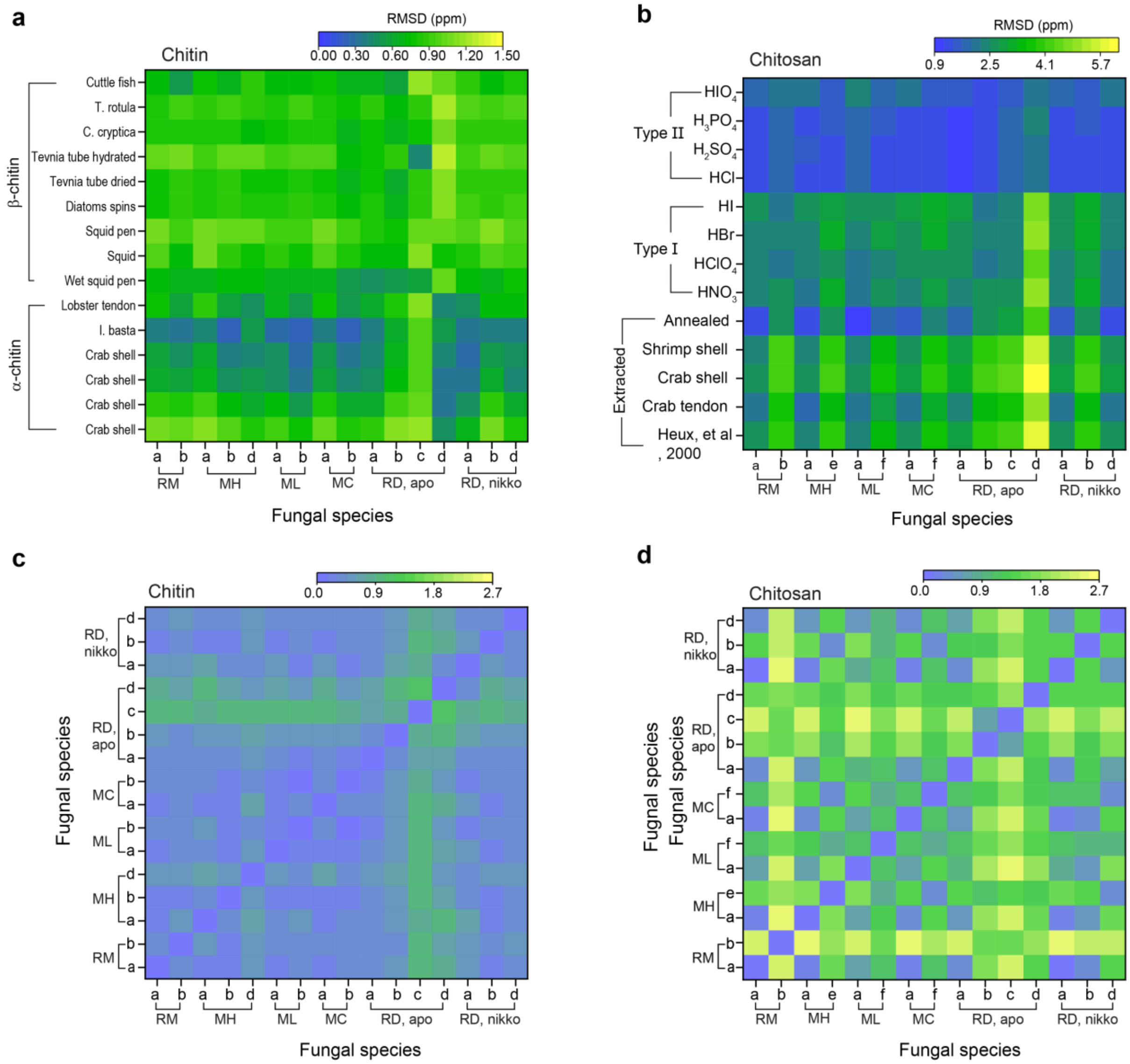

Supplementary Figure 24. Chemical shift comparison of differnt forms of chitin and chitosan. RMSDs of chemical shifts between literature-reported values of model forms with (**a**) chitin and (**b**) chitosan identified in different fungal species were plotted using heatmaps. Abbreviations for the fungal species: *R. sporus* (RM), *M. hiemalis* (MH), *M. lusitanicus* (ML), *M. circinelloides* (MC), apo-*R. delemar* (RD, apo), and nikkomycin-treated *R. delemar* (RD-nikko). RMSDs of chemical shifts are also calculated between different allomporphs identified in fungi for (**c**) chitin and (**d**) chitiosan. RMSDs are obtained using the following equation: $\mathbf{RMSD} = \sqrt{\frac{\sum_i^N (C_i^a - C_i^b)^2}{N}}$, where N is the number of carbon atoms (N=8 for chitin and N=6 for chitosan), i is for the specific types of carbon atom (e.g., C1 and C2) in chitin or chitosan, and a or b is for the serial number (e.g., Ch^a, Ch^b, Ch^c, Cs^a and Cs^b) of the subtypes of chitin or chitosan. Source data are provided in a Source Data file.

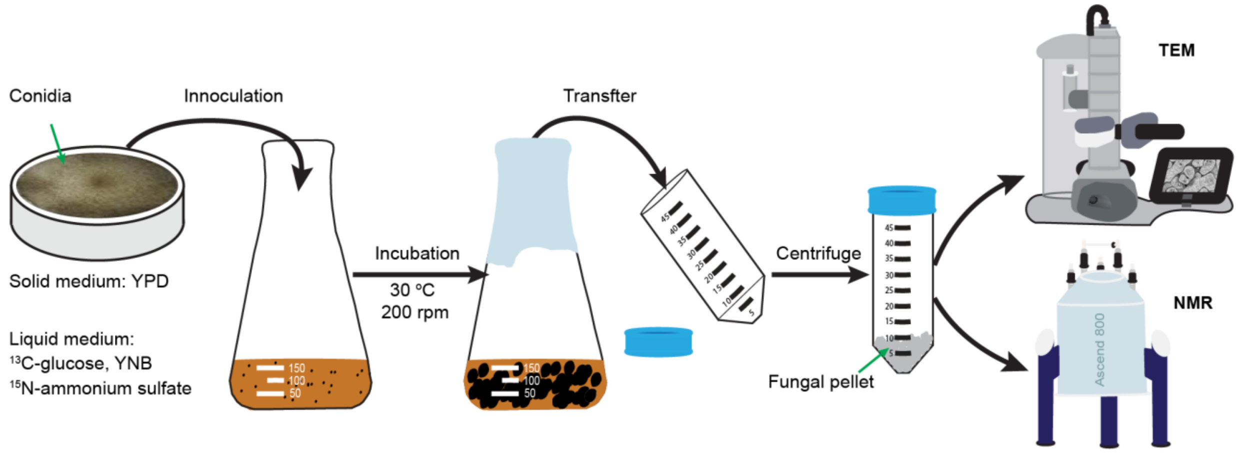

Supplementary Figure 25. Experimental flowchart of fungal cultivation. Initially, fungal conidia cultivated on YPD solid medium are inoculated into a 250 mL shake flask pre-filled with 100 ml of sterilized growth medium (^{13}C-glucose, ^{15}N-ammonium sulfate, and YNB). Subsequently, the shake flask is placed in a shaker for 5 days, maintaining a temperature of 30 ^{o}C and a shaking rate of 200 rpm. Following cultivation, the solution in the shake flask is transferred to 50 mL centrifuge flasks and centrifuged for 20 minutes at 6,500 rpm at 4 ^{o}C to collect the fungal pellets. The fungal pellets are thoroughly washed with phosphate-buffered saline (PBS) to eliminate any trace amounts of ^{13}C- or ^{15}N-labeled water-soluble fungal metabolites or source materials from the growth medium. The collected fungal pellets are stored in a freezer at -20 ^{o}C before TEM imaging or NMR measurements.

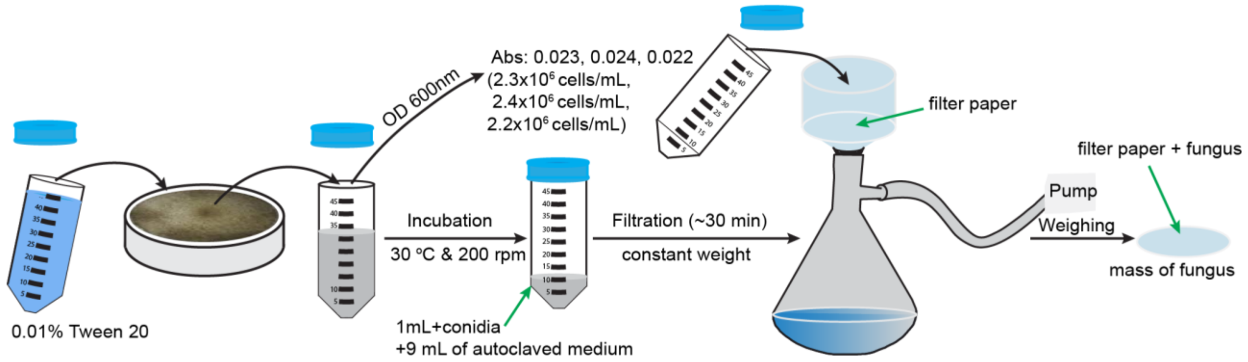

Supplementary Figure 26. Experimental flowchart of constructing fungal growth curves. First, fungal conidia are harvested using 0.01% Tween-20, and the optical density (OD) at 600 nm (with 0.01% Tween-20 solution) is measured. Second, 1 mL of the fungal conidia solution is transferred into a sterile 50 mL conical tube containing 9 mL of autoclaved growth medium. The tube is then placed in a cultivation shaker at 30 °C and 200 rpm. At designated time intervals of the cultivation period, the 50 mL conical tube is removed, and pump filtration is conducted for approximately 30 min to achieve a constant weight. Mass is determined at each specific time point during cultivation. All measurements are triplicated.

Supplementary Table 1. Estimation of the fraction of rigid and mobile molecules in *R. delemar*. Number of scans: NS.

R. delemar, apo						
Experiment	NS	Integral region	Integral	Mobile molecules % [a]	Rigid molecules % [b]	Dual detected % [c]
30s DP	96	110-50 ppm	6.92e9	76	33	9
2s DP	192	110-50 ppm	1.05e10			
Difference	/	110-50 ppm	3.39e9			
CP	128	110-50 ppm	1.41e10			
R. delemar, nikkomycin-treated						
30s DP	256	110-50 ppm	2.307e13	66	45	11
2s DP	256	110-50 ppm	1.528e13			
Difference	/	110-50 ppm	7.541e12			
CP	1024	110-50 ppm	9.140e13			

[a] The fraction of the mobile molecules were caldulated as ratio of spectral integrals of 2s DP over the 30s DP, with normalization by their correponding number of scans: $\mathbf{100\%} \times (\frac{\boldsymbol{Integral}^{2s\,DP}}{\boldsymbol{NS}^{2s\,DP}})/(\frac{\boldsymbol{Integral}^{30s\,DP}}{\boldsymbol{NS}^{30s\,DP}})$.

[b] To calcuate the fraction of the mobile molecules detected in CP-based spectrum, we overlay the CP and the difference spectrum to make sure that no peak in the difference spectrum is higher than that in CP (see **Supplmentary Fig. 11**) because CP detects some additional signals from some partially rigid molecules not covered by the difference spectrum. This yield a spectral scaling factor (S): 0.33 for apo sample and 0.109 for nikkomycin-treated sample. Then we obtain another ratio between the integrals of the scaled CP spectrum and the difference spectrum: 1.37 for the apo sample and 1.32 for the nikkomycin-treated sample, indicating that CP detects an additional 32-37% of signals not present in the difference spectrum. This ratio will be multiplied with the fraction of the difference spectrum relative to the original 30 s DP to get the final fraction of molecules detected by CP spectrum: $\frac{\boldsymbol{S} \times \boldsymbol{Integral}^{CP}}{\boldsymbol{Integral}^{Difference}} \times [\mathbf{1} - \left(\frac{\boldsymbol{Integral}^{2s\,DP}}{\boldsymbol{NS}^{2s\,DP}}\right)/\left(\frac{\boldsymbol{Integral}^{30s\,DP}}{\boldsymbol{NS}^{30s\,DP}}\right)] \times \mathbf{100\%}$

[c] Some molecules are detected simultaneously in 2s DP and CP, and their fraction is calculated as: Rigid molecules % + mobile molecules %-100%.

Supplementary Table 2. Relative abundances of rigid and mobile carbohydrate in the whole cell wall. The carbon percentage calculated from **Supplmentary Fig. 11** and **Supplementary Table 1** are highlighted in blue. The carbohdyrates within each domain are calculated using the fractions of these rigid/mobile domain and the respective fraction of a carbohydrate within each domain (**Supplementary Table 8**). Not detected (n.d.).

	R. delemar, apo	*R. delemar*, + nikkomycin
Rigid domain	33%	45%
Chitin	16.5%	30%
Chitosan	15%	15%
β-1,3-glucan	1.6%	n.d.
Mobile domain	76%	66%
Chitin	10.9%	1.7%
Chitosan	9.0%	6.2%
N-acetylgalactosamine	6.4%	0.6%
Galactosamine	5.7%	7.7%
Galactopyranose	14.6%	13.7%
Galfuranose	0.6%	0.3%
α-1,6-mannose	3.6%	2.5%
α-1,2-mannose	11.6%	9.8
β-1,3-glucan	3.6%	3.4%
α-1,3-fucose	7.9%	10.6%
Unknown	2.1%	9.6%

Supplementary Table 3. Measurements of the cell-wall thickness of *R. delemar*. All measurements are measured by image J and the unit is nm.[2] Reading x is abbreviated as Rx, e.g., R1 for Reading 1.

R. delemar, apo										
Reading	Cell 1	Cell 2	Cell 3	Cell 4	Cell 5	Cell 6	Cell 7	Cell 8	Cell 9	Cell 10
R1	129	118	156	146	116	101	113	132	101	143
R2	116	121	157	137	110	102	110	152	124	108
R3	100	117	125	139	123	105	117	131	102	104
R4	107	135	119	141	123	110	122	134	119	137
R5	110	131	139	121	104	97	135	95	94	155
R6	98	138	147	135	124	104	120	101	96	124
R7	102	131	129	119	138	122	109	108	106	132
R8	120	112	103	115	136	101	113	89	112	136
R9	104	102	116	144	111	100	135	104	109	134
R10	140	118	103	121	126	94	129	94	102	122
Average	113±13	122±11	129±19	132±11	121±10	104±7	120±9	114±20	107±9	129±15
Average for all readings	119±16									
R. delemar, + nikkomycin										
R1	226	181	173	197	196	191	251	246	265	224
R2	234	192	186	197	183	197	259	241	274	224
R3	226	176	168	213	202	183	214	237	268	212
R4	207	171	173	224	196	191	218	237	274	239
R5	194	172	192	208	205	195	236	250	276	228
R6	216	190	179	194	172	202	222	220	271	243
R7	231	171	185	188	183	188	235	257	262	228
R8	233	215	168	189	164	169	229	239	253	225
R9	216	202	196	176	199	192	251	239	241	222
R10	200	176	180	203	198	190	252	251	278	214
Average	218±13	185±14	180±9	199±13	190±13	190±9	237±15	241±10	266±11	226±9
Average for all readings	213±30									

Supplementary Table 4. Solid-state NMR experimental parameters for fungal cell wall characterization. T = sample temperature; B_0 = magnetic field; ν_{MAS} = MAS frequency; ns = number of scans; d_1 = recycle delay between scans; $t_{1,max}$ = maximum t_1 evolution time (for indirect dimension); $t_{1,inc}$ = increment for t_1 (for indirect dimension) evolution time; τ_{dw} = dwell time during direct FID acquisition; τ_{acq} = maximum acquisition time during direct FID detection; τ_{XY} = cross-polarization contact time during CP from channel X to channel Y; $\nu_{1H, dec}$ = dipolar decoupling field strength.

Experiment	T (K)	B_0 (T)	ν_{MAS} (kHz)	NS	d1 (s)	$t_{1, max}$ (ms)	$t_{1, inc}$ (μs)	τ_{dw} (μs)	τ_{acq} (ms)	τ_{HC} (ms)	τ_{mix} (ms)	$\nu_{1H\ dec}$ (kHz)	Samples
1D ^{13}C CP	290	18.8	13.5 or 15 [a]	256 or 1024	2			7/5	19.6/18	1		83 or 83	*R. delemar*, apo/+nikko or other mucor fungi
1D ^{13}C DP	290	18.8	13.5 or 15	1024	2			7/5	28.7/18			100 or 83	
1D ^{13}C DP	290	18.8	13.5 or 15	96 or 192	30 or 35								
2D CORD	290	18.8	13.5 or 15	16	1.6	7	25	7.5 or 5	18/14	1	53 τ_{CORD}	83 or 71	
2D CP J-INADEQUATE	290	18.8	13.5 or 15	16	1.5 or 2	8 or 7.5	60 or 22	7.5 or 5	18/14	0.5		92.5 or 71	*R. delemar*, apo and nikkomycin-treated
2D DP J-INADEQUATE	290	18.8	13.5 or 15	16	1.5 or 2	10 or 7.5	20 or 22	7.5 or 5	19/14			83	
2D water-edited	280	9.4	15	256 or 64	2	5.5	50	8	16	1	50 τ_{PDSD}	83	
1D Torchia ^{13}C-T_1	298	9.4	10 or 15	256	2			10/8	14/16	1	50 τ_{PDSD}	71 or 83	
2D PDSD	273	18.8	15	64	2	6.5	25	5	14	1	1000 τ_{PDSD}	83	
2D PAR	290	18.8	13.5	64	1.7	5.7	27	7.5	16	0.5	15 τ_{PAR}	83	*R. delemar*, apo
2D ^{15}N-^{1}H HETCOR	290	18.8	13.5	8	1.6	2.3	41	7.5	18			92.5	
2D N(CA)CX with DARR	290	18.8	13.5	32	1.7	10	100	7.5	18		100 τ_{DARR}	92.5	
2D hCH	302	18.8	60	16	2	4.26	33.33	8.5	19.9	0.2 for CP1&2		10	
2D hNH	302	18.8	60	16	2	6.4	50.12	15.3	19.9	2 for CP1, 0.2 or 2 for CP2		10	
2DhChH (RFDR)	302	14.1	60	32	2.5	4.26	33.33	5	20	0.2 for CP1, 0.05 for CP2	1.67	10 for sltTPPM; 15 for WALTZ-16	14.1

[a] 13.5 kHz for *R. delemar* apo sample except 1s-PDSD (15 kHz and 273 K) and 2D water-edited experiments (15 kHz and 280 K). 15 kHz for nikkomycin-treated *R. delemar* sample and other *Rhizopus* and *Mucor* species.

Supplementary Table 5. ^{13}C and ^{15}N chemical shifts of rigid polysaccharides in *R. delemar* cell walls. Superscripts are used to denote different allomorphs. Not applicable (/). Unidentified (-). Minor forms (m).

Carbohydrate		C1	C2	C3	C4	C5	C6	CO	CH_3	N	Experiments	References
***R. delemar*, apo**												
chitin	a	104.2	55.3	74.0	83.3	75.7	60.5	174.5	22.9/22.7	123.8	^{13}C-^{13}C CORD, ^{13}C CP J-INADEQUATE	Kang et al., 2018 and Fernando et al., 2021[3-4]
	b	104.3	55.3	74.1	84.3	75.1	60.7	174.9	22.9	123.7		
	c	104.3	55.4	74.0	83.1	75.1	58.9	174.9	23.7	123.9		
	d(m)	104.3	54.9	73.2	83.4	76.2	60.9	173.1	22.4	123.6		
chitosan	a	101.9	56.0	72.6	79.5	75.0	60.7/60.3	/	/	32.6		
	b	98.1	56.5	71.1	79.8/77.0	75.3	60.7	/	/	33.6		
	c(m)	97.3	57.8	70.5	80.0	75.2	60.8	/	/	33.6		
	d	100.7	55.4	70.0	78.8	74.7	61.6	/	/	33.6		
β-1,3-glucan		103.7	74.4	86.8	68.2	77.4	61.3	/	/	/		
***R. delemar*, + nikkomycin**												
chitin	a	104.4	55.2	73.6	83.2	76.3	60.5	173.9	22.8	-	^{13}C-^{13}C CORD, ^{13}C CP J-INADEQUATE	Chakraborty et al., 2021[1]
	b	104.0	55.6	73.9	83.5	76.4	61.1	174.9	22.9	-		
	d	103.7	54.9	73.5	83.4	76.0	60.8	174.6	23.1	-		
chitosan	a	102.2	56.1	72.8	79.9	75.3	60.8	/	/	-		
	d(m)	101.6	56.0	72.7	80.5	75.4	61.6	/	/	-		
	b	99.8	57.0	72.4	77.6	75.6	61.0	/	/	-		

Supplementary Table 6. ^{13}C chemical shifts of mobile polysaccharides in *R. delemar* cell walls. Superscripts are used to denote different allomorphs. Not applicable (/). Unidentified (-).

***R. delemar*, apo**

Carbohydrate		C1	C2	C3	C4	C5	C6
Ch	a&b	104.1	55.3	73.4	83.1	74.7	60.1
	d	104.3	54.7	73.2	82.9	74.9	61.0
Cs	a	101.8	56.5	72.6	77.9	75.3	60.8
	b	98.0	56.6	72.6	77.9	75.3	60.8
GalNAc		95.4	57.4	72.4	82.4	75.9	61.1
GalN		91.3	54.5	69.2	77.9	-	-
Mn^{1,2}	a	101.0	78.9	70.7	67.5	73.8	61.7
	b	99.3	80.4	-	-	-	-
	c	99.3	79.0	-	-	-	-
Mn^{1,6}		102.3	70.6	73.7	67.6	71.2	66.2
β-1,3-glucan	a	103.3	73.9	-	-	-	-
	b	103.6	74.5	-	-	-	-
α-fucose	a	90.0	74.3	71.1	71.1	67.7	19.6
	b	96.7	74.8	77.1	70.2	68.4	20.2
	c	91.0	74.5	70.2	73.3	66.6	20.1
	d	94.5	71.9	72.3	69.7	67.1	19.2
	e	98.3	67.0	70.3	71.4	67.4	15.9
Gal	a	102.5	/	/	/	/	/
	b	103.9	71.0	72.8	70.3	76.7	61.3
	c	99.9	70.7	73.2	70.1	71.5	63.3
	d	99.7	72.6	/	/	/	/
	e	101.0	70.6	/	/	/	/
Gal*f*		107.3	90.5	/	/	/	/
Unknown	a	-	-	71.3	85.9	62.4	-
	b	-	-	70.0	85.0	61.5	-
uA*	a	102.7	52.7	-	-	-	-
	b	101.5	55.6	74.4	83.9	-	-
	c	89.8	55.2	73.4	83.2	75.4	60.7
uN*	a	98.8	54.3	69.2	77.9	-	-
	b	93.4	57.5	74.6	80.0	-	-

***R. delemar*, + Nikkomycin**

Carbohydrate		C1	C2	C3	C4	C5	C6	Reference
Ch	a&b	104.2	55.5	-	-	-	-	Chakraborty et al., 2021[1]
Cs	a	102.2	56.0	72.8	79.8	75.7	61.3	
	d	99.8	57.1	72.5	78.0	75.6	61.1	
Mn^{1,2}	a	101.3	79.3	71.0	67.8	74.1	62.0	
	b	99.6	79.4	-	-	-	-	
	c	98.9	79.4	-	-	-	-	
Mn^{1,6}		102.8	71.0	73.7	67.5	71.7	66.3	
β-1,3-glucan	a	102.5	73.6	82.9	-	-	-	
	b	103.7	74.0	85.7	-	-	-	
α-fucose	a	90.1	74.5	-	-	-	-	Furevi et al., 2022[5]
	b	96.7	75.2	76.6	70.6	68.8	20.4	
	e	98.5	69.2	73.8	70.1	69.7	15.9	
	f	93.0	72.2	70.4	69.2	68.0	19.9	
	g	90.0	76.4	72.9	73.3	68.0	19.9	
	h	90.7	76.5	73.8	71.8	68.0	14.4	
Gal	b	104.2	73.4	-	-	76.8	61.9	Kang et al., 2018[4]
	c	100.2	70.8	68.0	74.1	70.4	63.7	Fernando et al., 2023[6]
	d	100.6	72.7	73.7	70.6	76.9	61.7	
	e	101.4	70.1	-	-	-	-	
Galf		107.7	86.0	-	-	-	-	
Unknown	a	-	-	70.6	85.0	62.1	-	
	b	-	-	71.4	86.2	62.5	-	
	c	88.0	76.6	-	-	-	-	
	d	88.5	77.1	-	-	-	-	
uN*		99.9	57.1	72.6	78.0	75.6	61.1	
uA*		99.1	55.0	-	81.4	-	-	

* Note: Unknown amino sugars without (uN) and with (uA) acetyl groups are categorized empirically by their C4 chemical shifts (> 81 ppm for uA; < 81 ppm for uN).

Supplementary Table 7. Chemical shifts of amino acids in *R. delemar* cells. Superscripts are used to represent the same type of amino acids (AAs). Not applicable (/). Unidentified (-). The rigid AAs are identified by 2D 53 ms ^{13}C-^{13}C CORD (CORD) spectra and the mobile AAs are assigned using the 2D ^{13}C DP J-based INADEQAUTE (DP-INADEQUATE).

***R. delemar*, apo**

Amino acids		Cα	Cβ	Cγ	Cδ	Cε	CO	CO'
**Rigid AAs (CORD)**								
Aspartic	D	50.8	40.1	/	/	/	171.7	174.6
Arginine	R	52.0	30.5	25.3	44.0	/	172.1	/
Isoleucine	I	58.0	40.0	25.6(15.5)	12.4	/	171.6	/
Leucine	L	55.7	42.6	29.7	25.2	/	171.7	/
Lysine	K	53.3	30.9	28.1	25.4	42.1	172.3	/
Threonine	T	56.0	60.7	22.7	/	/	174.7	/
Valine	V	58.1	33.1	19.1	/	/	172.4	/
**Mobile AAs (DP-INADEQUATE)**								
Alanine	A^a	50.2	17.4	/	/	/	174.9	/
	A^b	49.7	17.3	/	/	/	174.9	/
Asparagine	N	51.2	37.0	/	/	/	172.3	176.7
Glycine	G	43.1	/	/	/	/	172.0	/
Histidine	H	56.0	28.8	124.5/128.3	-	-	171.2	/
Phenylalanine	F^a	55.9	43.0	132.1	-	-	172.5	/
	F^b	54.3	40.3	134.6	-	-	171.9	/
	F^c	52.9	40.2	136.1	-	-	171.3	/
	F^d	55.0	42.8	135.9	-	-	169.6	/
Proline	P^a	60.4	30.3	25.2	48.5	/	172.6	/
	P^b	59.3	30.7	24.3	47.3	/	169.2	/

***R. delemar*, + nikkomycin**

Amino acids		Cα	Cβ	Cγ	Cδ	Cε	CO	CO'
**Rigid AAs (CORD)**								
Alanine	A^a	51.0	17.5	/	/	/	173.2	/
	A^b	49.2	21.4	/	/	/	173.3	/
Arginine	R	52.3	32.2	25.7	44.1	/	172.5	/
Isoleucine	I	58.3	39.5	27.0(15.9)	12.8	/	172.9	/
Aspartic	D	56.1	40.3	/	/	/	172.6	176.9
Lysine	K	52.9	34.9	31.5	25.0	40.2	173.0	/
Glutamic	E	55.9	32.9	37.2	/	/	172.8	176.2
Valine	V	60.2	31.1	19.8	/	/	173.8	/
Threonine	T	56.1	61.4	23.3	/	/	173.0	/
**Mobile AAs (DP-INADEQUATE)**								
Alanine	A^a	52.0	17.3	/	/	/	171.4	/
	A^b	50.2	17.6	/	/	/	171.4	/
	A^c	50.9	17.0	/	/	/	175.5	/
	A^d	52.4	17.8	/	/	/	173.6	/
Phenylalanine	F^a	54.4	41.0	136.2	-	-	170.3	/
	F^b	54.8	40.6	134.8	-	-	174.1	/
	F^c	51.3	37.6	139.6	-	-	177.3	/
	F^d	56.6	413	134.8	-	-	172.2	/
Asparagine	N	52.5	39.5	/	/	/	174.0	178.7
Glycine	G	43.6	/	/	/	/	171.3	/
Phenylalanine	F^e	57.4	37.2	136.4	-	-	173.5	/
Proline	P^a	61.4	30.1	25.5	48.9	/	175.0	/
	P^b	60.5	31.0	25.9	48.7	/	173.9/175.1	/
Tryptophan	W^a	55.0	28.4	130.6	-	-	175.0	/
	W^b	55.7	28.4	130.7	-	-	175.8	/
	W^c	54.5	28.5	129.9	-	-	176.5	/
Valine	V	56.4	29.2	19.2	/	/	178.7	/

Supplementary Table 8. Molar composition of polysaccharides in cell walls. The molar percentages of rigid cell-wall polysaccharides are estimated using integrals (volume) of cross peaks in 2D ^{13}C-^{13}C 53-ms CORD. The molar percentages of mobile cell-wall polysaccharides are estimated by using well-resolved peaks (C1 and C2) of 2D J-based ^{13}C-^{13}C DP-INADEQUATE. All errors are propagated from at least 3 individual integrals of well-resolved peaks for each type of molecule.

Rigid domain			
Polysaccharide	Chitin%	Chitosan%	β-1,3-glucan
R. delemar, apo	50 ± 3	45 ± 7	5 ± 1
R. delemar, + nikko	66 ± 3	34 ± 3	/
R. microsporus	76 ± 6	24 ± 5	/
M. lusitanicus	73 ± 5	27 ± 4	/
M. circinelloides	65 ± 8	35 ± 9	/
M. hiemalis	68 ± 7	32 ± 13	/

Mobile domain				
	R. delemar, apo		*R. delemar*, +nikko	
Polysacharide	%	Peak	%	Peak
Chitin	14.3	C1, C2	2.5	C1, C2
Chitosan	11.9	C1, C2	9.4	C1, C2
N-acetylgalactosamine	8.4	C1, C2	0.9	C1, C2
Galactosamine	7.5	C1, C2	11.7	C1, C2
Galactopyranose	19.2	C1, C2	20.8	C1, C2
Galfuranose	0.8	C1, C2	0.5	C1
α-1,6-mannose	4.7	C1, C2	3.8	C1, C2
α-1,2-mannose	15.3	C1, C2	14.8	C1, C2
β-1,3-glucan	4.7	C1, C2	5.1	C1, C2
α-1,3-fucose	10.4	C1, C2	16	C1, C2
Unknown	2.8	C3, C4 and C5	14.6	C3, C4, C5, C1 and C2

The following peaks were used for the estimation of molar composition of each rigid polysaccharide in fungal cell walls: *R. delemar*: $Ch^{a,b}$-C4-1, C1-4, C4-5+C4-3, C5-4+C3-4; Ch^{c}-C1-6, C6-1, C6-2 and C2-6; Ch^{d}-ChCO-1, ChCO-4, ChCO-5, ChCO-3, ChCO-2, ChCO-6; Cs^{a}-C1-5, C1-3, C1-2 and C4-3; Cs^{b}-C1-5, C1-3, C1-2, C3-6 and C4-6; Cs^{c}-C1-5, C1-3, C1-2 and C1-4; Cs^{d}-C1-5, C1-3, C1-2 and C1-6; B-C1-4, C1-5, C5-4, C2-4, C3-5, C3-2 and C3-4. *R. delemar* + nikko: $Ch^{a,b}$-C4-1, C2-4, C6-2 and C5-4; Ch^{d}-ChCO-1, ChCO-4, ChCO-5 and ChCO-6; Cs^{a}-C3-4, C5-4, C4-6 and C4-3; Cs^{b}-C3-4, C4-6, C5-4 and C1-2; Cs^{d}-C4-1, C5-1, C3-1 and C2-1. *R. microspores*: $Ch^{a,b}$-C4-1, C1-4, C1-5, and C5-4; Cs^{a}-C3-1, C4-1, C3-4, C5-4, and C4-5; Cs^{b}-C2-1, C1-2, C3-5 and C4-5. *M. lusitanicus*: $Ch^{a,b}$-C4-1, C1-4, C5-4 and C4-5; Cs^{a}-C3-4, C5-4, C4-5 and C3-1; Cs^{f}-C4-2, C3-4, C2-1 and C1-2. *M. cirinelloides*: $Ch^{a,b}$-C4-1, C1-4, C2-1 and C6-5; Cs^{a}-C3-4, C5-4, C4-5 and C4-3; Cs^{f}-C3-4, C4-3, C4-5 and C2-1. *M. hiemalis*: $Ch^{a,b}$-C4-1, C1-4, C5-1, C4-6 and C4-2; Ch^{d}-ChCO-1, ChCO-4, ChCO-3 and ChCO-2. Cs^{a}-C2-1, C1-2, C5-4 and C3-4; Cs^{e}-C2-1, C1-2, C3-4 and C5-4.

Supplementary Table 9. Intermolecular cross peaks in apo and nikkomycin-treated *R. delemar*. The chemical shifts for the two dimensions of the spectra (ω_1 and ω_2). Asterisks indicate interactions where only one peak is observed along the diagonal line with the absence of the other.

***R. delemar*, apo**		
Interaction	ω_1 or ω_2 (ppm)	ω_1 or ω_2 (ppm)
**Chitin-chitosan**		
Ch^{a,b}Me-Cs^{a}1	22.9	101.9
Ch^{a,b}Me-Cs^{b}1	22.9	98.1
Ch^{a,b}Me-Cs^{d}1	22.9	100.7
Ch^{a,b}Me-Cs4	22.9	79.7
Cs^{a}3-Ch^{a,b}Me*	72.6	22.9
Cs^{b}3-Ch^{a,b}Me*	71.1	22.9
Ch^{a,b}Me-Cs3*	22.9	70.7
Ch^{a,b}Me-Cs^{b}2	22.9	56.5
Ch2-Cs^{b}1	55.3	98.1
Ch^{c}6-Cs^{b}1	58.9	98.1
Ch^{a,b}6-Cs^{a}1	60.5	101.9
Ch^{a,b}6-Cs^{b}1	60.5	98.1
Ch^{a,b}6-Cs^{d}1	60.5	100.7
Ch^{c}6-Cs4*	58.9	79.5
Ch2-Cs4	55.3	79.5
Cs3-Ch^{a,c}4	70.6	83.3
Cs3-Ch^{b}4	70.6	84.3
Ch3-Cs^{b}1	74.1	98.1
Ch3-Cs^{a}1	74.1	101.9
Ch^{a,c}4-Cs^{a}1	83.3	101.9
Ch^{b}4-Cs^{a}1	84.3	101.9
Ch1-Cs4	104.2	79.5
Ch^{a,c}4-Cs^{d}1	83.3	100.7
Ch^{b}4-Cs^{d}1	84.3	100.7
Cs^{b}1-Ch1	98.1	104.2
Cs^{a}1-Ch1	101.9	104.2
Cs^{d}1-Ch1	100.7	104.2
Cs^{b}3-ChCO	71.1	174.9
Cs^{a}3-ChCO	72.6	174.9
Cs^{a}1-ChCO	101.9	174.9
Cs^{b}1-ChCO	98.1	174.9
**Chitin-Chitin**		
Ch^{a}6-Ch^{b}4	60.5	84.3
Ch^{a,b}Me-Ch^{c}6	22.9	60.5
Ch^{c}6-Ch^{a,b}6	58.9	60.5
**Chitosan-Chitosan**		
Cs^{b}1-Cs^{a}1	98.1	101.9
Cs^{b}6-Cs^{a}1	60.7	101.9
**Chitin-β-1,3-glucan**		
Ch^{a,b}Me-B5	22.9	77.4
Ch^{c}6-B5	58.9	77.4
B5-ChCO	77.4	174.9
B4-Ch^{a,c}5	68.2	75.7
**Chitosan-β-1,3-glucan**		
B5-Cs^{a}1	77.4	101.9
B5-Cs^{b}1	77.4	98.1
B5-Cs^{d}1	77.4	100.7
B4-Cs^{a}1*	68.2	101.9
B4-Cs^{b}1*	68.2	98.1
B4-Cs4*	68.2	79.5
**Carbohydrates-Protein**		
Iγ2-Cs^{a}1*	15.5	101.9
Iγ2-Cs^{b}1*	15.5	98.4
Iγ2-Cs4*	15.5	79.5
Iγ2-Cs^{a}5*	15.5	75.0
Iγ2-Ch3*	15.5	74.0
Iγ2-Ch5*	15.5	75.1
Iγ1-Ch1	25.6	104.2
Iγ1-Ch3	25.6	74.0

***R. delemar*, + nikko**		
Interaction	ω_1 or ω_2 (ppm)	ω_1 or ω_2 (ppm)
**Chitin-chitosan**		
ChMe-Cs^{a}1	22.8	102.2
ChMe-Cs^{d}1	22.8	101.6
ChMe-Cs^{b}1	22.8	99.8
ChMe-Cs^{a,b}2	22.8	57
ChMe-Cs^{a,b}3	22.8	72.4
ChMe-Cs^{a}4	22.8	79.9
ChMe-Cs^{b}4	22.8	77.6
ChMe-Cs^{a,b}6	22.8	60.8
Ch1-Cs^{a}1	104.4	102.2
Ch1-Cs^{d}1	104.4	101.6
Ch1-Cs^{b}1	104.4	99.8
Ch1-Cs^{b}2	104.4	57.2
Ch1-Cs^{b}3	104.4	72.4
Ch2-Cs^{a}1	55.2	102.2
Ch2-Cs^{b}1	55.2	57.0
Ch3-Cs^{b}1	83.2	99.8
Ch4-Cs^{a}1	83.2	102.2
Ch4-Cs^{b}1	83.2	99.8
Ch4-Cs^{a,b}5	83.2	75.3
Ch4-Cs^{a}3*	83.2	72.8
Ch4-Cs^{b}3*	83.2	72.4
Cs^{a,b}3-Ch4*	72.4	83.2
Ch5-Cs^{b}1	76.3	102.2
Ch6-Cs^{b}1	60.5	99.8
ChCO-Cs^{a}1	174.8	102.2
ChCO-Cs^{d}1	174.8	101.6
ChCO-Cs^{b}1	174.8	99.8
ChCO-Cs^{a,b}3	174.8	72.4
ChCO-Cs^{a}4	174.8	79.9
ChCO-Cs^{b}4	174.8	77.6
Ch4-Cs^{b}2	83.2	57.0
Ch1-Cs^{a}4	104.4	79.9
Ch5-Cs^{a}1	76.3	102.2
**Chitosan-chitosan**		
Cs^{a}2-Cs^{b}1	56.1	99.8
Cs^{a}4-Cs^{b}1	79.9	99.8
Cs^{a}4-Cs^{b}3	79.9	72.4
Cs^{a}5-Cs^{b}1	75.3	99.8
Cs^{a}1-Cs^{b}1	102.2	99.8
Cs^{a}1-Cs^{b}2	102.2	57
**Carbohydrates-Protein**		
Iγ1-Ch1	25.8	104.4
Iγ1-Ch4	25.8	83.3
Iγ1-Ch5	25.8	76.4
Iγ1-Ch3	25.8	73.7
Iγ1-Ch6*	25.8	61.0
Cs^{b}3-Iγ2*	15.9	72.4
Cs^{b}2-Iγ2*	15.9	57.0

Supplementary Table 10. Water-edited intensities of polysaccharide carbon sites. The intensity ratios were calculated by comparing the peak intensity in water-edited and control spectra, which were normalized by the number of scans. The error bars represent the standard deviations propagated from NMR signal-to-noise ratios.

Cross peaks	apo	+ nikko	Cross peaks	apo	Cross peaks	+ Nikko	Cross peaks	apo	Cross peaks	+ Nikko
**Average Ch[a,b]**	**0.24**	**0.36**	**Average Cs[a]**	**0.59**	**Average Cs[a]**	**0.50**	**Average Cs[b]**	**0.47**	**Average Cs[b]**	**0.49**
Ch1-1	0.17±0.04	0.35±0.01	Cs1-1	0.86±0.06	Cs1-1	0.42±0.04	Cs1-1	0.58±0.10	Cs1-1	0.39±0.02
Ch1-4	0.15±0.04	0.33±0.03	Cs1-2	0.38±0.05	Cs1-2	0.52±0.06	Cs1-2	0.28±0.05	Cs1-4	0.58±0.05
Ch1-5	0.17±0.03	0.37±0.03	Cs1-3	0.55±0.05	Cs1-3	0.57±0.05	Cs1-3	0.62±0.06	Cs1-5	0.55±0.07
Ch1-3	0.18±0.03	0.37±0.02	Cs1-4	0.78±0.06	Cs1-4	0.61±0.07	Cs1-4	0.56±0.03	Cs1-3	0.63±0.06
Ch1-6	0.20±0.05	0.43±0.07	Cs1-5	0.78±0.05	Cs1-5	0.60±0.05	Cs1-5	-	Cs1-2	0.48±0.06
Ch1-2	0.17±0.02	0.34±0.02	Cs1-6	0.44±0.02	Cs1-6	-	Cs1-6	0.45±0.10	Cs4-1	0.55±0.08
Ch4-1	0.14±0.03	0.34±0.05	Cs5-1	0.55±0.04	Cs3-1	0.58±0.09	Cs4-1	0.35±0.06	Cs4-4	0.46±0.03
Ch4-4	0.28±0.04	0.34±0.03	Cs5-2	0.54±0.03	Cs3-4	0.43±0.05	Cs4-2	0.39±0.04	Cs4-5	0.40±0.08
Ch4-5	0.21±0.03	0.31±0.04	Cs5-3	0.39±0.06	Cs3-5	0.49±0.04	Cs4-3	0.36±0.02	Cs4-3	0.38±0.07
Ch4-3	0.25±0.07	0.33±0.04	Cs5-4	0.66±0.03	Cs3-3	0.51±0.07	Cs4-4	0.59±0.05	Cs4-2	0.46±0.13
Ch4-6	0.20±0.02	0.32±0.07	Cs5-5	0.41±0.01	Cs3-2	-	Cs4-5	0.48±0.06	-	-
Ch4-2	0.30±0.04	0.36±0.05	Cs5-6	0.45±0.03	Cs2-1	0.54±0.10	Cs4-6	0.52±0.08	Cs5-1	0.45±0.11
Ch2-1	0.18±0.02	0.31±0.02	Cs6-1	0.87±0.04	Cs2-4	0.45±0.09	Cs5-1	0.40±0.03	Cs5-4	0.45±0.08
Ch2-4	0.29±0.03	0.35±0.04	Cs6-2	0.61±0.05	Cs2-5	0.47±0.06	Cs5-2	0.44±0.03	Cs5-5	0.45±0.03
Ch2-5	0.28±0.02	0.37±0.02	Cs6-3	0.40±0.04	Cs2-3	0.45±0.06	Cs5-3	0.37±0.01	Cs5-3	0.42±0.15
Ch2-3	0.21±0.02	0.33±0.01	Cs6-4	0.81±0.10	Cs2-2	0.45±0.04	Cs5-4	0.65±0.04	Cs5-2	0.46±0.10
Ch2-6	0.21±0.03	0.34±0.04	Cs6-5	0.62±0.08	-	-	Cs5-5	0.24±0.01	-	-
Ch2-2	0.27±0.03	0.37±0.02	Cs6-6	0.60±0.07	-	-	Cs5-6	0.43±0.04	Cs2-1	0.46±0.07
Ch3-1	0.20±0.03	0.31±0.02	Cs4-1	-	Cs4-1	0.44±0.06	Cs2-1	0.39±0.03	Cs2-4	0.46±0.08
Ch3-4	0.27±0.06	0.35±0.02	Cs4-2	-	Cs4-2	0.55±0.08	Cs2-2	0.59±0.09	Cs2-5	0.81±0.13
Ch3-5	0.41±0.03	0.43±0.01	Cs4-3	-	Cs4-3	0.43±0.04	Cs2-3	0.52±0.09	Cs2-3	0.49±0.13
Ch3-3	0.50±0.04	0.42±0.01	Cs4-4	-	Cs4-4	0.52±0.03	Cs2-4	0.65±0.06	Cs2-2	0.47±0.05
Ch3-6	0.37±0.03	0.54±0.03	Cs4-5	-	Cs4-5	0.48±0.05	Cs2-5	0.47±0.06	-	-
Ch3-2	0.18±0.02	0.37±0.02	Cs4-6	-	Cs4-6	-	Cs2-6	0.48±0.03	-	-

Cross peaks	apo	Cross peaks	apo	Cross peaks	apo
**Average Ch[c]**	**0.27**	**Average Cs[c]**	**0.71**	**Average β-1,3-glucan**	**0.29**
Ch1-Ch1	0.17±0.04	Cs1-1	0.83±0.07	B3-1	0.31±0.04
Ch1-Ch4	0.24±0.03	Cs1-2	0.66±0.12	B3-2	0.13±0.04
Ch1-Ch3	0.22±0.03	Cs1-3	0.89±0.17	B3-3	0.28±0.04
Ch1-Ch6	0.50±0.07	Cs1-4	0.84±0.08	B3-4	0.16±0.03
Ch1-Ch2	0.18±0.06	Cs1-5	0.56±0.08	B3-5	0.12±0.07
Ch4-Ch1	0.34±0.06	Cs5-1	0.62±0.07	B5-1	0.24±0.09
Ch4-Ch4	0.28±0.03	Cs5-2	0.64±0.05	B5-2	0.33±0.04
Ch4-Ch3	0.27±0.05	Cs5-3	0.38±0.05	B5-3	0.41±0.08
Ch4-Ch6	0.33±0.11	Cs5-4	0.74±0.09	B5-4	0.45±0.06
Ch4-Ch2	0.25±0.07	Cs5-5	0.39±0.03	B5-5	0.44±0.08
Ch2-Ch1	0.18±0.02	Cs3-1	0.91±0.05	B4-1	0.14±0.02
Ch2-Ch4	0.26±0.07	Cs3-2	0.69±0.05	B4-2	0.22±0.07
Ch2-Ch3	0.30±0.06	Cs3-3	0.88±0.06	B4-3	0.31±0.02
Ch2-Ch6	0.24±0.07	Cs3-4	0.89±0.08	B4-4	0.35±0.04
-	-	Cs3-5	0.73±0.04	B4-5	0.40±0.04

Supplementary Table 11. ^{13}C-T_1 and ^{1}H-$T_{1\rho}$ relaxation time constants and dipolar order parameters. Single exponential equations were used to fit the observed de cay: $I(t) = e^{-t/T_1}$. The fit parameters were obtained, and the standard deviations of these parameters were calculated as error bars. Average values are shown in bold.

***R. delemar*, apo**				
Carbohydrate		Chemical shifts	^{13}C-T_1 Time (s)	^{1}H-$T_{1\rho}$ Time (ms)
chitin		**Average**	**1.66**	**5.55**
	a,b	104.0	2.3 ±0.1	8.9±0.4
	b	84.0	2.2 ±0.2	7.3±0.6
	a,c	83.3	2.4 ±0.1	7.9±0.5
	a,c	75.0	1.1±0.1	2.7±0.3
	a	60.5	0.9±0.1	3.0±0.2
	c	59.0	1.3±0.1	3.4±0.2
	a,b,c	55.5	1.5±0.1	5.8±0.5
chitosan		**Average**	**0.74**	**1.86**
	a	102.0	0.84±0.06	1.41±0.05
	b	98.1	0.63±0.03	1.30±0.05
	a,b	79.5	0.61±0.05	1.53±0.07
	a	72.6	0.95±0.09	3.1±0.2
	b	71.2	0.68±0.05	1.99±0.09
β-1,3-glucan		**Average**	**0.65**	**1.83**
		77.5	0.51±0.03	1.30±0.07
		68.1	0.66±0.05	1.58±0.08
		61.3	0.8±0.1	2.6±0.2

***R. delemar*, nikko**				
chitin		**Average**	**2.64**	**12.0**
	a,b	104.2	4.0±0.2	17.5±0.3
	a,b	83.3	4.0±0.2	16.3±0.4
	a,b	73.6	2.2±0.3	10.7±0.5
	a,b	60.9	1.0±0.2	6.8±0.4
	a,b	55.4	2.1±0.2	9.0±0.5
chitosan		**Average**	**1.10**	**5.1**
	a	102.2	1.04±0.09	4.3±0.2
	a	79.9	0.93±0.07	3.8±0.3
	a	72.8	1.6±0.2	8.3±0.5
	a	75.3	1.7±0.2	8.3±0.5
	b	99.8	0.80±0.04	3.2±0.2
	b	77.6	0.84±0.06	4.1±0.2
	b	72.4	0.9±0.1	4.7±0.4
	b	57.0	0.99±0.08	4.4±0.2

Supplementary Table 12. ^{13}C and ^{15}N chemical shifts of rigid polysaccharides in other Rhizopus and Mucor species. The chemical shifts of apo and nikkomycin-treated *R. delemar* samples are excluded as they have been reported earlier in **Supplementary Table 5**. Superscripts are used to denote different allomorphs.

R. microsporus	C1	C2	C3	C4	C5	C6	CO	Me
Ch^a	104.0	55.5	73.6	83.8	76.1	61.2	175.0	22.9
Ch^b	104.2	55.6	73.9	84.0	75.8	61.4	174.4	23.1
Cs^a	102.0	56.2	72.9	80.2	75.4	60.9		
Cs^b	98.4	56.9	70.4	80.2	71.9	62.1		
M. hiemalis								
Ch^a	104.0	55.5	73.7	83.3	76.0	61.1	175.3	23.3
Ch^b	104.1	55.5	73.6	83.7	76.2	61.3	174.6	22.9
Ch^d	104.4	55.3	73.5	84.2	76.2	60.4	173.8	22.8
Cs^a	102.4	56.3	73.0	80.6	75.3	60.8		
Cs^e	99.4	57.0	71.9	78.0	75.4	61.6		
M. lusitanicus								
Ch^a	104.0	55.4	73.5	83.7	75.8	61.2	174.8	22.6
$Ch^{a,b}$	104.3	55.3	73.6	83.4	76.0	60.9	174.2	22.7
Cs^a	102.1	56.2	73.2	80.8	74.9	62.4		
Cs^e	100.6	57.0/60.0	72.5	79.0	74.0	62.0		
M. circinelloides								
Ch^a	104.1	55.4	73.6	83.4	76.1	61.0	175.1	22.7
Ch^b	104.5	55.3	73.7	83.4	76.0	61.0	174.4	22.8
Cs^a	102.2	56.1	73.1	79.8	75.3	61.2		
Cs^f	100.3	57.1	72.3	78.2	75.6	61.3		

Supplementary References

1. Chakraborty, A.; Fernando, L. D.; Fang, W.; Dickwella Widanage, M. C.; Wei, P.; Jin, C.; Fontaine, T.; Latgé, J.-P.; Wang, T., A molecular vision of fungal cell wall organization by functional genomics and solid-state NMR. *Nature Communications* 2021, *12*, 6346.
2. Schneider, C. A.; Rasband, W. S.; Eliceiri, K. W., NIH Image to ImageJ: 25 years of image analysis. *Nature Methods* 2012, *9*, 671-675.
3. Fernando, L. D.; Dickwella Widanage, M. C.; Penfield, J.; Lipton, A. S.; Washton, N.; Latgé, J.-P.; Wang, P.; Zhang, L.; Wang, T., Structural polymorphism of chitin and chitosan in fungal cell walls from solid-state NMR and principal component analysis. *Frontiers in Molecular Biosciences* 2021, *8*, 727053.
4. Kang, X.; Kirui, A.; Muszyński, A.; Widanage, M. C. D.; Chen, A.; Azadi, P.; Wang, P.; Mentink-Vigier, F.; Wang, T., Molecular architecture of fungal cell walls revealed by solid-state NMR. *Nature Communications* 2018, *9*, 2747.
5. Furevi, A.; Ruda, A.; Angles d'Ortoli, T.; Mobarak, H.; Ståhle, J.; Hamark, C.; Fontana, C.; Engström, O.; Apostolica, P.; Widmalm, G., Complete ^{1}H and ^{13}C NMR chemical shift assignments of mono-to tetrasaccharides as basis for NMR chemical shift predictions of oligo- and polysaccharides using the computer program CASPER. *Carbohydrate Research* 2022, *513*, 108528.
6. Fernando, L. D.; Pérez-Llano, Y.; Dickwella Widanage, M. C.; Jacob, A.; Martínez-Ávila, L.; Lipton, A. S.; Gunde-Cimerman, N.; Latgé, J.-P.; Batista-García, R. A.; Wang, T., Structural adaptation of fungal cell wall in hypersaline environment. *Nature Communications* 2023, *14*, 7082.
